# Differential transcriptional responses to Ebola and Marburg virus infection in bat and human cells

**DOI:** 10.1038/srep34589

**Published:** 2016-10-07

**Authors:** Martin Hölzer, Verena Krähling, Fabian Amman, Emanuel Barth, Stephan H. Bernhart, Victor A. O. Carmelo, Maximilian Collatz, Gero Doose, Florian Eggenhofer, Jan Ewald, Jörg Fallmann, Lasse M. Feldhahn, Markus Fricke, Juliane Gebauer, Andreas J. Gruber, Franziska Hufsky, Henrike Indrischek, Sabina Kanton, Jörg Linde, Nelly Mostajo, Roman Ochsenreiter, Konstantin Riege, Lorena Rivarola-Duarte, Abdullah H. Sahyoun, Sita J. Saunders, Stefan E. Seemann, Andrea Tanzer, Bertram Vogel, Stefanie Wehner, Michael T. Wolfinger, Rolf Backofen, Jan Gorodkin, Ivo Grosse, Ivo Hofacker, Steve Hoffmann, Christoph Kaleta, Peter F. Stadler, Stephan Becker, Manja Marz

**Affiliations:** 1 RNA Bioinformatics and High Throughput Analysis, Faculty of Mathematics and Computer Science, Friedrich Schiller University Jena, Leutragraben 1, 07743, Jena, Germany; 2 Institute of Virology, Philipps University Marburg, Hans-Meerwein-Str. 2, 35043 Marburg, Germany; 3 German Center for Infection Research (DZIF), partner site Gießen-Marburg-Langen, Hans-Meerwein Str. 2, 35043, Marburg, Germany; 4 Bioinformatics Group, Department of Computer Science, University of Leipzig, Härtelstraße 16-18, 04107, Leipzig, Germany; 5 FLI Leibniz Institute for Age Research, Beutenbergstraße 11, 07745 Jena, Germany; 6 Transcriptome Bioinformatics, Junior Research Group, Leipzig Research Center for Civilization Diseases, University of Leipzig, Härtelstraße 16-18, 04107, Leipzig, Germany; 7 Center for non-coding RNA in Technology and Health, University of Copenhagen, Grønnegårdsvej 3, 1870, Frederiksberg C, Denmark; 8 Department of Veterinary Clinical and Animal Sciences, University of Copenhagen, Grønnegårdsvej 3, 1870, Frederiksberg C, Denmark; 9 Theoretical Biochemistry Group, Institute of Theoretical Chemistry, University of Vienna, Währingerstraße 17, 1090, Vienna, Austria; 10 Bioinformatics Group, Department of Computer Science, University of Freiburg, Georges-Köhler-Allee 106, 79110, Freiburg, Germany; 11 Research Group Theoretical Systems Biology, Department of Bioinformatics, Friedrich Schiller University Jena, Ernst-Abbe-Platz 2, 07743, Jena, Germany; 12 Institute of Computer Science, Martin-Luther University Halle-Wittenberg, Von-Seckendorff-Platz 1, 06120, Halle/Saale, Germany; 13 Department of Soil Ecology, UFZ - Helmholtz Centre for Environmental Research, Theodor-Lieser-Str. 4, 06120, Halle/Saale, Germany; 14 German Centre for Integrative Biodiversity Research (iDiv) Halle-Jena-Leipzig, Deutscher Platz 5e, 04103, Leipzig, Germany; 15 Biozentrum, University of Basel, Klingelbergstraße 50/70, CH-4056, Basel, Switzerland; 16 Chair of Bioinformatics, Faculty of Mathematics and Computer Science, Friedrich Schiller University Jena, Ernst-Abbe-Platz 2, 07743, Jena, Germany; 17 Junior Professorship for Computational EvoDevo, Bioinformatics, Department of Computer Science, University of Leipzig, Härtelstraße 16-18, 04107, Leipzig, Germany; 18 TFome Research Group, Bioinformatics Group, Interdisciplinary Center of Bioinformatics, Department of Computer Science, University of Leipzig, Härtelstraße 16-18, 04107, Leipzig, Germany; 19 Paul-Flechsig-Institute for Brain Research, University of Leipzig, Jahnallee 54, 04109, Leipzig, Germany; 20 Leibniz Institute for Natural Product Research and Infection Biology Hans Knöll Institute (HKI), Systems Biology and Bioinformatics, Beutenbergstraße 11a, 07745, Jena, Germany; 21 Department of Bioanalytical Ecotoxicology, UFZ-Helmholtz Centre for Environmental Research, Leipzig, Germany; 22 Doctoral School of Science and Technology, AZM Center for Biotechnology Research, Lebanese University, Tripoli, Lebanon; 23 TRON - Translational Oncology at the University Medical Center of the Johannes Gutenberg University Mainz gGmbH, Mainz, Germany; 24 Institute of Aquaculture, University of Stirling, Stirling FK9 4LA, Scotland, U.K; 25 Medical University of Vienna, Center for Anatomy and Cell Biology, Währingerstraße 13, 1090, Vienna, Austria; 26 Centre for Biological Signalling Studies (BIOSS), University of Freiburg, Freiburg, Germany; 27 Research group Bioinformatics and Computational Biology, Faculty of Computer Science, University of Vienna, Währingerstraße 29, 1090, Vienna, Austria; 28 Research Group Medical Systems Biology, Institute for Experimental Medicine, Christian-Albrechts-University Kiel, Brunswiker Str. 10, 24105, Kiel, Germany

## Abstract

The unprecedented outbreak of Ebola in West Africa resulted in over 28,000 cases and 11,000 deaths, underlining the need for a better understanding of the biology of this highly pathogenic virus to develop specific counter strategies. Two filoviruses, the Ebola and Marburg viruses, result in a severe and often fatal infection in humans. However, bats are natural hosts and survive filovirus infections without obvious symptoms. The molecular basis of this striking difference in the response to filovirus infections is not well understood. We report a systematic overview of differentially expressed genes, activity motifs and pathways in human and bat cells infected with the Ebola and Marburg viruses, and we demonstrate that the replication of filoviruses is more rapid in human cells than in bat cells. We also found that the most strongly regulated genes upon filovirus infection are chemokine ligands and transcription factors. We observed a strong induction of the JAK/STAT pathway, of several genes encoding inhibitors of MAP kinases (*DUSP* genes) and of *PPP1R15A*, which is involved in ER stress-induced cell death. We used comparative transcriptomics to provide a data resource that can be used to identify cellular responses that might allow bats to survive filovirus infections.

An Ebola virus (EBOV) outbreak in West Africa of unprecedented severity resulted in over 28,600 cases and 11,300 deaths as of April 2016 (http://www.cdc.gov/vhf/ebola/outbreaks/2014-west-africa/previous-case-counts.html). EBOV and Marburg virus (MARV) are closely related filoviruses, with a nucleotide identity of 49.5%. They contain single-stranded RNA genomes with a negative orientation that are approximately 19 kb in size and encode seven structural proteins^1^. The natural hosts of filoviruses are presumed to be bats^2^, which may be the origin of the recent EBOV outbreak in West Africa^3^. *Rousettus aegyptiacus* is the natural reservoir of MARV^4^,^5^,^6^, and it survives MARV infections without any signs of the disease^7^,^8^. However, humans with filovirus infections experience a severe fever and vascular leakage, with high fatality rates^9^. Surprisingly little is known about the response of human cells to EBOV and MARV infections, and the response in bat cells has not been investigated at all. Barrenas *et al*.^10^ used a next-generation sequencing approach to understand the cellular immune response of vaccinated cynomolgus macaques after an EBOV challenge. Microarray-based studies have described the differential expression of several known cellular genes after EBOV infections in mice and rhesus monkeys^11^,^12^,^13^,^14^. However, the overall response of human and bat cells to filovirus infections is not yet known. Key proteins in the infection process and their regulatory circuits have not been defined, and the transcriptional landscape of non-coding RNAs (ncRNAs) and alternative mRNA isoforms is unexplored. Cellular targets are an attractive alternative for the development of antiviral drugs because viruses cannot adapt to a change in the host cell as easily as they can develop a resistance to an antiviral drug over the course of treatment^15^,^16^. To establish effective antiviral strategies, it is necessary to understand how the infected cells respond to filovirus infections and how this response differs between bats and humans. We explored the cellular regulatory response mechanisms by sequencing the full transcriptomes of immortalized cells of human and bat origin at three different time points post infection (p.i.). We constructed a full *de novo* transcriptome assembly (see Materials and Methods) based on the RNA-Seq data of *R. aegyptiacus*.

Here, we provide a systematic report on (1) a genome-wide analysis of EBOV, MARV, human, and bat transcripts, as well as (2) single genes that show strong differential regulation, (3) the regulatory transcription factors, and (4) the corresponding pathways that are involved in the response to EBOV and MARV infections. We focus on the transcriptional differences and similarities between EBOV- and MARV-infected human and bat cells.

## Materials and Methods

### Cells and RNA extractions

**Figure 1:**
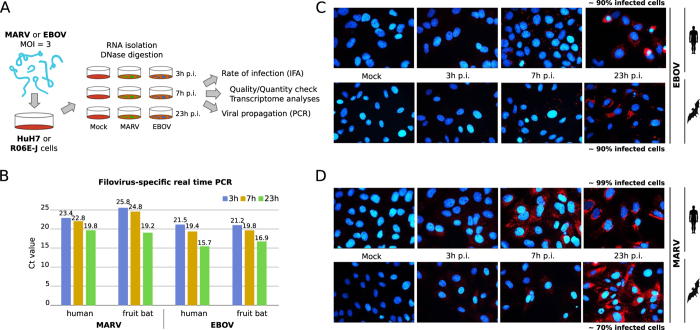
Monitoring sample preparation. (**A**) HuH7 and R06E-J cells were infected with MARV or EBOV (MOI = 3) or left uninfected (Mock). Samples were collected after 3, 7 and 23 h post infection (p.i.). (**B**) RNA of infected and uninfected cells was isolated at 3, 7 and 23 h p.i., checked for quality and quantity, and filovirus-specific real time PCR was performed according to Panning *et al*.^21^. (**C**,**D**) To determine the number of infected cells, immunofluorescence analyses were performed with infected cells grown on one coverslip within each well used for RNA preparation. Infected cells were visualized (red) using mouse monoclonal antibodies against EBOV (**C**) or MARV (**D**) nucleoproteins and fluorescently tagged secondary antibodies. DAPI staining was used to visualize cell nuclei (blue). Ct-cycle threshold.

**Figure 2:**
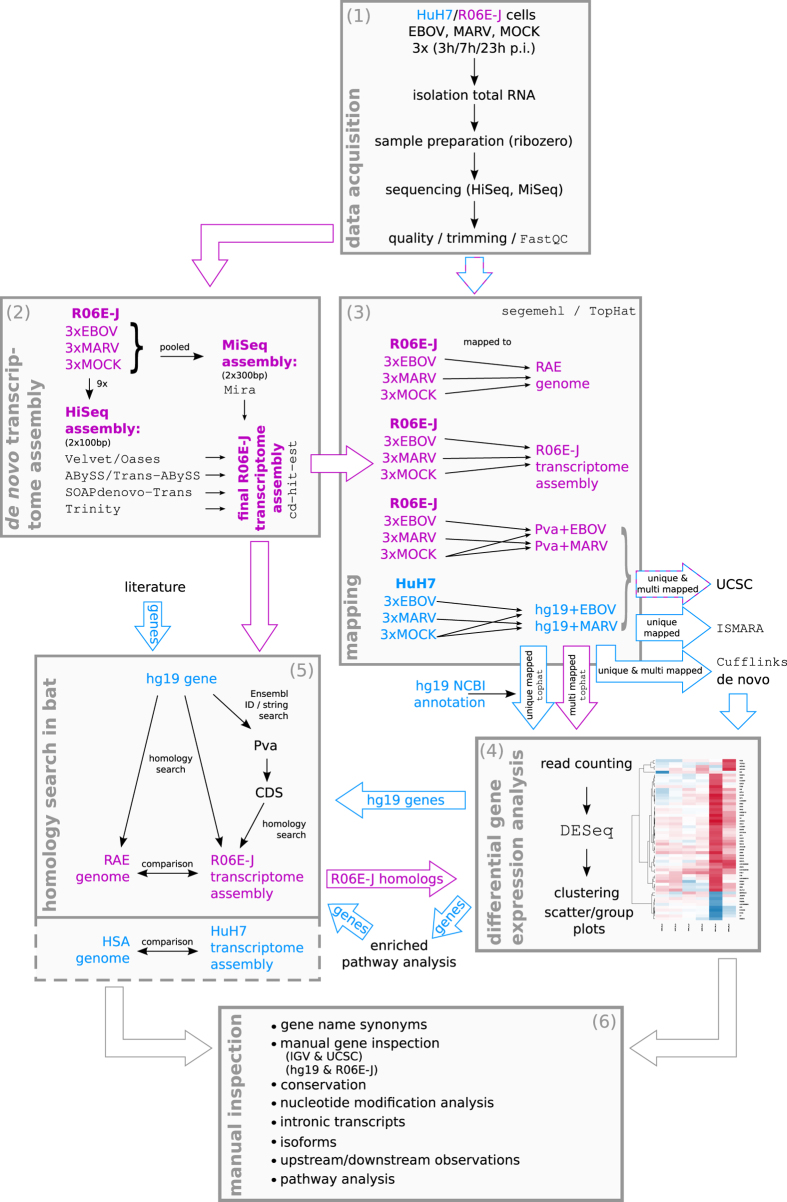
Methods pipeline. (1) **Data acquisition:** Total RNA from HuH7 and R06E-J cell lines 3, 7 and 23 h p.i. was depleted of ribosomal RNA and sequenced. We controlled the quality and trimmed the data with PrinSeq and FastQC. (2) For bat RNA, we assembled a ***de novo*** **transcriptome** by adding pooled MiSeq to HiSeq data using various assembly tools and parameter settings. (3) **Mapping** was performed for Mock-, EBOV-, and MARV-treated cells onto human/bat genomes and the bat transcriptome with segemehl and TopHat. (4) **Differential gene expression analysis** was performed by counting uniquely mapped reads and applying a DESeq analysis. The results were used for clustering and scatter/group plot analyses. (5) **Homology searches in bats** were performed for all significantly differentially expressed genes from (4) and for the genes that were presumed to be involved in the response to infection based on the literature and an enriched pathway analysis. The *R. aegyptiacus* genome and coding sequences from *P. vampyrus* were used to validate and detect homologous sequences in the bat transcriptome. Detected homologs were used for the differential gene-expression analysis. We also investigated the quality of the transcriptome assembly by comparing the human and *R. aegyptiacus* genomes with the corresponding assembly. (6) During the **manual inspection**, we identified the synonyms of gene names and noted their existence in the relevant pathways. Each candidate gene was manually investigated in the IGV and UCSC browsers for the human and bat samples from all time points. We report the conservation of genes according to the 100 Species Vertebrate Multiz Alignment to chimp, mouse, dog, elephant and chicken sequences. We searched for nucleotide modifications (differential SNPs, posttranscriptional modifications), intronic transcripts and regulators, alternative splicing and isoforms, and upstream and downstream transcript characteristics.

**Figure 3:**
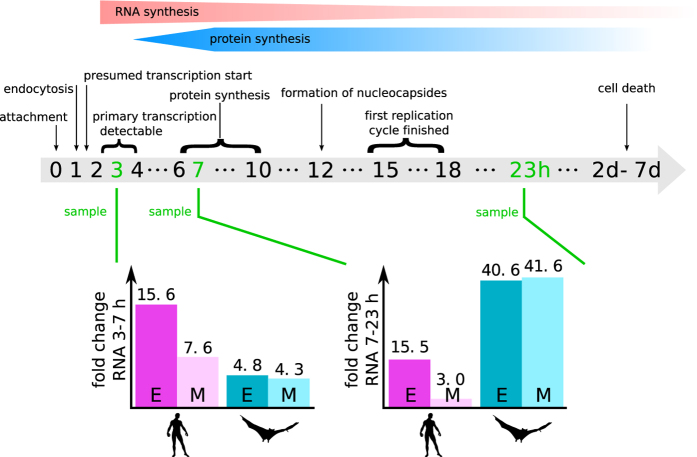
Human HuH7 cells support an earlier onset of filoviral RNA synthesis than bat R06E-J cells. Attachment of the virions to the cell surface leads to cell mediated macropinocytosis^93^. Within one hour after attachment to the host cell, Ebola is endocytosed^94^, viral transcription can be detected at 2–4 h p.i.^95^, and newly synthesized proteins can be detected via IFA at 6–10 h p.i. Mature nucleocapsids primed for transport are present at 10–12 h p.i. (Materials and Methods). The first replication cycle is finished after 15–18 h, when virions are released from the host cells. Host cells die between 2 to 7 days. Between 3 and 7 h p.i. of EBOV-infected R06E-J cells, we observed an ~4.8X increase in the number of reads that mapped uniquely to the EBOV genome. This indicates that EBOV genes are rapidly replicated and transcribed in the bat cells in the first 4 h p.i. (see Table S2 for normalized read counts). We observed a further 41X increase in reads between 7 and 23 h p.i. in R06E-J cells. So, this RNA synthesis rate slows down within this next 16 h (compared to 4.8X^4^ ≃ 530X). In comparison, unique reads mapping to the EBOV genome in HuH7 cells increased 15.6X between 3 and 7 h p.i. and a further 15.5X in the following 16 h. This result indicates a significant increase in the RNA synthesis rate of viral RNAs in the first few hours and a marked decrease in the following hours.

We infected 4 × 10^5 ^HuH7 cells^17^ (a human hepatoma cell line) and 4 × 10^5 ^R06E-J cells^18^ (an embryonic cell line from *R. aegyptiacus*) with EBOV (Ebola virus strain Zaire, Mayinga, GenBank: NC_002549) or MARV (Lake Victoria Marburg virus, Leiden, GenBank: JN408064.1^19^) at a multiplicity of infection (MOI) of three (Fig. 1A). We used HuH7 cells because EBOV infections in humans induce the majority of their histopathological features in the liver, and these cells are highly susceptible to filovirus infections^20^. We used immortalized cells because primary cells from bats (macrophages and dendritic cells) are not available in the large quantities we required for our RNA-Seq analyses (9 samples from each cell type). Our analyses consisted of computational and extensive manual investigations as shown in Fig. 2. At 3, 7 and 23 h p.i., cells were harvested, and total RNA was isolated using an RNeasy Mini Kit (QIAGEN) according to the manufacturer's instructions. These time points correspond to the different stages of the viral replication cycle (Fig. 3). Replication and transcription take place after 3 h, proteins are produced at 7 h, which may regulate further transcription, and a complete replication cycle occurs after 23 h. DNaseI digestion was performed. At each time point, RNA was also isolated from non-infected (Mock) control cells. Quality controls were performed to ensure proper infection rates and viral propagation. Real-time PCR was used to detect filovirus RNA (polymerase genes)^21^ and to demonstrate the amplification of viral RNA over the time course of the infections (Fig. 1B). Ct-values are inversely proportional to the amount RNA detected, and these values for the MARV-infected HuH7 cells at 3 and 7 h p.i. were lower than the values for the MARV-infected R06E-J cells. The Ct-values for the EBOV-infected cells showed no clear difference. An immunofluorescence analysis (IFA) of the cells was performed using mouse monoclonal antibodies directed against nucleoproteins of EBOV (B6C5, 1:20) and MARV (59-9-10, 1:100). An anti-mouse secondary antibody coupled with Alexa 594 (1:500) was used to detect these viral nucleoproteins, and DAPI (4′,6′-diamidino-2-phenylindole) staining was used to visualize cell nuclei (1 *mg*/*ml*, 1:2000). IFAs of the MARV and EBOV nucleoproteins revealed that a MOI of 3 was sufficient to initially infect a high percentage of cells (90% of human and bat cells infected with EBOV, 70% of bat cells and 99% of human cells infected with MARV, Fig. 1C/D). The quantity and quality of the RNA was assessed using a NanoDrop spectrophotometer and an Agilent Bioanalyzer. Nine samples at different time points were generated from human cells (HuH7-Mock-3h, HuH7-Mock-7h, HuH7-Mock-23h, HuH7-EBOV-3h, HuH7-EBOV-7h, HuH7-EBOV-23h, HuH7-MARV-3h, HuH7-MARV-7h, HuH7-MARV-23h) and bat (R06E-J-Mock-3h, R06E-J-Mock-7h, R06E-J-Mock-23h, R06E-J-EBOV-3h, R06E-J-EBOV-7h, R06E-J-EBOV-23h, R06E-J-MARV-3h, R06E-J-MARV-7h, R06E-J-MARV-23h).

### Sample preparation and sequencing

The total RNA of the 18 samples was shipped to LGC Genomics for the construction of cDNA libraries. Ribo-Zero was used for rRNA depletion, and the Illumina TruSeq kit was used for library construction. Illumina sequencing was performed in a 2 × 100 nt paired-end mode on a HiSeq 2000 system. R06E-J cells were stimulated with interferons, PolyIC or thapsigargin to mimic the induction of the interferon system or a stress response by the endoplasmic reticulum (ER) of the cells. Prior to stimulation, R06E-J cells were examined for interferon competence via a vesicular stomatitis virus (VSV) bioassay. The cells secreted cytokines after PolyIC transfection, and those cytokines partially protected R06E-J cells from VSV infection (data not shown). RNA was isolated from these cells, pooled with the 9 previously mentioned *R. aegyptiacus* cell samples and shipped to GATC Biotech for normalization and sequencing on an Illumina MiSeq system (2 × 300 nt mode). This library of longer paired-end reads was used to improve the *de novo* transcriptome assembly of *R. aegyptiacus*. All reads were preprocessed based on their Phred quality score. At the 3′ end, bases with a quality score <20, a 5′-bias and poly-A tails were removed with PRINSEQ-lite^22^ (v0.20.3). Quality was assessed and controlled before and after processing with FastQC^23^ (v0.10.1).

### Genome and annotation data

The human genome GRCh37/hg19 was downloaded from the UCSC^24^ ftp server (ftp://hgdownload.cse.ucsc.edu/goldenPath/hg19/). The annotation data were obtained from the NCBI (GRCh37 patch release 5) and Ensembl (GRCh37 release 75). The genomic sequence of *Pteropus vampyrus* (Pva, GCA_000151845.1), the closest related species to *R. aegyptiacus* (both Megachiroptera) and with well established annotation files, was downloaded from the UCSC site (ftp://hgdownload.cse.ucsc.edu/goldenPath/pteVam1/) and used for the homology search. The genome sequence of *R. aegyptiacus* was published in early 2016 by the Boston University School of Medicine. We used all scaffolds and the corresponding annotation data downloaded from the NCBI database (ftp://ftp.ncbi.nlm.nih.gov/genomes/Rousettus_aegyptiacus/) for mapping and differential gene expression analysis. The genomic sequence and annotation data for the Zaire Ebola virus (KM034562.1) were extracted from the UCSC Ebola Genome Portal (ftp://hgdownload.cse.ucsc.edu/goldenPath/eboVir3/), which is based on the 2014 West African outbreak^25^. Genome and annotation data for the Lake Victoria Marburg virus Leiden (JN408064.1) were obtained from the NCBI-GenBank database.

### *De novo* transcriptome assemblies

The nine HiSeq libraries for *H. sapiens* and *R. aegyptiacus* underwent quality control assessments and were used for transcriptome assembly (see electronic supplement, Table ES1A). Long reads of the pooled MiSeq libraries were included in the assembly process for *R. aegyptiacus*. For the bat HiSeq libraries, 372,082,040 paired-end reads were assembled *de novo* with Velvet^26^ (v1.2.10), followed by Oases^27^ (v0.2.08), the ABySS/TransABySS^28^,^29^ pipeline (v1.5.1/v1.4.8), SOAPDenovo-Trans^30^ (v1.0.3) and Trinity^31^ (v20131110) using default parameters and multiple k-mer values (25/35/45/55/65/75). *R. aegyptiacus*-derived MiSeq paired-end reads (38,028,488) were preprocessed and assembled using Mira^32^ (v4.0.0). The resulting contigs from each assembly tool were merged together and clustered based on sequence similarities using CD-HIT-EST^33^ (-c 0.95, v4.6) to improve the quality of the final assembly. The final *R. aegyptiacus* assembly contained 977,787 contigs (human: 986,920 contigs), which is similar to the results of Lee *et al*.^34^. Of these, 277,595 contigs had a length greater than 1,000 bp. The bat assembly had a maximum contig length of 36,073 bp with an N50 of 3,923. We used QUAST^35^ (v2.3) to calculate several statistics for the independent and merged transcriptome assemblies (Table ES1B).

### A comparison between the genome and *de novo* transcriptome assemblies of humans and bats

To assess the quality of our *de novo* transcriptome assemblies, we used various read count thresholds over all mapped HuH7 and R06E-J samples (Table S3) to extract transcript subsets from the *H. sapiens* and *R. aegyptiacus* genomes, respectively. We denoted these filtered subsets as expressed and blasted (E-value < 10^−10^) them against the *de novo* transcriptome assemblies of the human or bat cells. We defined a transcript (derived from the genomic sequence) as valid, and therefore correctly assembled, if we obtained a minimum of one blast hit with an alignment length >90% of the query. For the human transcriptome assembly, we found between 93.0% and 98.1% of the expressed transcripts, and for *R. aegyptiacus* 81.3–94.0%. Therefore, the transcriptome assemblies were of sufficient quality. The results for different transcript subsets are shown in Table S3. Most of the missing transcripts can be explained by a low read coverage in comparison to the length of the transcript or a non-uniform distribution of reads along the transcript. These transcripts may be assembled as partial contigs (alignment length ≤90%). The higher number of valid transcripts derived from the human genome can be explained by its better annotation and assembly status compared to that of the relatively new *R. aegyptiacus* genome at the scaffold level.

### Genome and transcriptome mapping

RNA-Seq data for the HuH7 and R06E-J samples were mapped to the concatenated virus-host genome file (for each combination of the two viruses and three genomes/transcriptomes) in the following two ways: (1) using TopHat^36^ (v2.0.11) with the default parameters and (2) using segemehl^37^ (v0.1.9) with the split read option -S. The indexing and sorting of the SAM files was performed using samtools (v0.1.19). The ViennaNGS^38^ toolbox (v0.10) was used for processing and visualization of the mapped RNA-Seq data. Uniquely mapped reads aligned by TopHat were used for all statistical calculations and differential gene-expression analyses. The segemehl mappings were used to support the detailed analyses of special splice variants because of the tool’s capability for mapping multi-split reads. The read mapping rates and statistics can be found in Table S1. Although we observed some differences for some genes (e.g., *NPC1*) regarding their read counts between the *R. aegyptiacus* genome and transcriptome assembly, the calculated fold changes used in the downstream analyses differed only slightly among all genes.

### *De novo* transcript annotation

We used Cufflinks^39^ (v2.2.1) in *de novo* mode to predict transcript loci based on the TopHat mappings of all HuH7 samples. Cuffmerge was used to combine the Cufflinks-assembled transcripts into a final transcriptome assembly of 18,391 locations for the human genome.

### Differential gene expression analysis

For a differential gene expression analysis of hg19, we used reads of Mock, EBOV and MARV samples uniquely mapped by TopHat together with the gene annotation data from the NCBI. Raw read counting was performed using BEDTools^40^ (v2.21.0) multicov with the split option. The resulting read counts were directly passed to DESeq^41^ in the R/Bioconductor package (v2.14). Due to the lack of replicates, we performed pairwise comparisons between all of the different infection conditions and time points with a false discovery rate of 0.1. To compare the characteristics between HuH7 and R06E-J cells at 3, 7 and 23 h p.i., we used the EBOV and MARV samples at the identical time points as replicates in the DESeq analysis (*padj *≤ 0.1). In addition to using gene annotations from the NCBI, we processed the *de novo* gene loci obtained from Cufflinks in an identical manner. Furthermore, we used Cuffquant and Cuffnorm to calculate FPKM values for each locus from the Cuffmerge -G results. In addition to the normalized read-count-based method, we calculated the maximum read peak for each gene and sample using BEDTools genomecov (-d -split). We used the notation “=” when the difference between the two samples was <15%, “↑/↓” when there was up to a two-fold difference, and “n↑” when the difference was up to *n*-fold (greater than 2-fold).

### Clustering of differentially expressed genes

Fold changes in gene expression were calculated by adding a pseudocount of 1 to the raw read counts of each gene. Differentially expressed genes in infected cells, compared to their expression in their Mock control cells, were determined using the DESeq package as described above, and only genes with *padj* <0.1 were considered differentially expressed for a particular contrast (infection versus Mock). For the clustering of fold changes in gene expression, the log2-fold change (FC) for each gene *g* was calculated as log2(*g*_*infection*_) − log2(*g*_*mock*_). The log2-fold changes in the expression of genes having a log2-fold change greater than five in at least one of the contrasts were visualized via hierarchical clustering. Euclidean distances and the Ward method for agglomeration were used (Figure ES2A).

### Identification of co-regulated genes

To compare the differential gene expression for the Mock/EBOV and Mock/MARV treatments in HuH7 and R06E-J cells, log2-fold changes, as computed by DESeq, were visualized using scatter plots in R (x-value: FC of Mock/EBOV; y-value: FC of Mock/MARV). The scatterplots were overlaid with contour plots for a two-dimensional kernel estimate (kde2d; MASS package) using the default parameters. Outliers are labeled with their respective gene names in the electronic supplement (Figure ES4B).

### Search for human-gene homologs within bat transcripts

To compare human and bat genes, we defined homologous loci between human genes and the transcripts in the *R. aegyptiacus* assembly. A direct comparison between human genes and *R. aegyptiacus* transcripts led to several unidentified orthologous pairs. To improve ortholog detection, we used the annotated orthologs of *P. vampyrus* from Ensembl to act as an intermediate between the two species. Sequence comparisons were performed using BLASTn+ (v.2.2.27+), and hits under the restrictive E-value threshold of <10^−50^ were considered orthologs. We defined homologous genes between the human and recently published *R. aegyptiacus* genomes.

### Comprehensive gene analysis

We obtained a comprehensive set of candidate genes based on more than 600 significantly differentially expressed genes identified by the DESeq analysis combined with ~900 genes extracted from a literature search and a review of immune system-related pathways. We manually investigated differential gene expression, changes in expression profiles, nucleotide changes, conservation in other species, intron-exon structures, alternatively spliced isoforms, intronic transcripts and 5′-UTR/3′-UTR discrepancies with the help of the UCSC and IGV^42^ (v2.3.39) browsers. Each gene, its synonyms, functional information, screenshots of genomic locations, isoforms, fold changes and maximum read counts are listed in the electronic supplement (nine samples per species).

### Pathway enrichment

We examined which of the differentially expressed genes were over-represented in KEGG database^43^ pathways. Gene set enrichment analyses were performed using a hypergeometric test. FDR-corrected p-values were significant at 0.05. We set the threshold to a value of *p* < 0.1 using a hypergeometric test and FDR corrections^44^. The evaluation was performed with the R-package GAGE^45^ to obtain KEGG pathway information and with pathview^46^ to allow for visualization.

### Motif activity response analysis

A motif activity response analysis with MARA was performed as previously described^47^ using the reads uniquely mapped in TopHat as discussed above. We performed MARA on the EBOV and MARV samples separately to obtain independent motif rankings and target predictions for each virus. We also performed one analysis that considered all samples at once to compare the motif activity changes observed after EBOV and MARV infections. The activities obtained from this analysis were used to create the activity change comparison plots. To relate the activity of a motif *m* in virus-infected cells (*A*_*m*_,_*Virus*_) to its activity in the corresponding Mock control (*A*_*m*_,_*Mock*_), the activity change (*A*_*m*_,_Δ_) was calculated as follows:
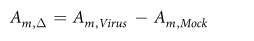
with the error calculated as:
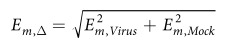


### The fast-track, *in-silico* “Fight against Ebola”

After the start of the 2014 outbreak, we decided to speed up our analysis, which otherwise would have taken up to three years. To accomplish this, 40 scientists with experience in analyzing RNA-Seq data came together to “Fight against Ebola” and manually investigated each gene. During this “hackathon”, we investigated 1,500 genes (7.5% of human protein-coding genes). Each gene was analyzed in detail using the IGV and UCSC browsers. The gene, its synonyms, functional information, screenshots of genomic locations, isoforms, fold changes and maximum read counts can be found in the electronic supplement (http://www.rna.uni-jena.de/supplements/filovirus_human_bat/igo.php).

### qRT-PCR

We repeated the infection of HuH7 and R06E-J cells and isolated RNA for qRT-PCR analyses. An IFA of these cells (Figure S7E) revealed that the infection rates were lower than in the previous experiment used for RNA-Seq. Only ~40% (~70%) of the R06E-J cells were infected with EBOV (MARV) in comparison to ~90% (~70%) in the first experiment (Fig. 1C). The infection rates were only slightly lower for HuH7 cells (Figure S7E). RNA was isolated and reverse transcribed with random hexamers. For qRT-PCR analyses of *NPC1* and *TLR3* gene expression, degenerate primers that amplified both the human and the bat mRNA sequences were used. *HAVCR1* gene expression was measured using human- and bat-specific primers. Expression values were normalized to 18S rRNA levels. All primer sequences are listed in File ES7A. qPCRs were performed on an Applied Biosystems 7500 Real-Time PCR System using SYBR Green chemistry (iTaq mix, BioRad) according to the manufacturer’s instructions. Mean values from triplicate analyses were calculated and quantified with the system’s built-in software according to standard curves constructed for each amplicon from serial cDNA dilutions. Relative quantification values, melting curves and agarose gel pictures for the three genes are presented in the electronic supplement, Section ES7.

## Results and Discussion

### Differences in early viral replication velocities

**Figure 4:**
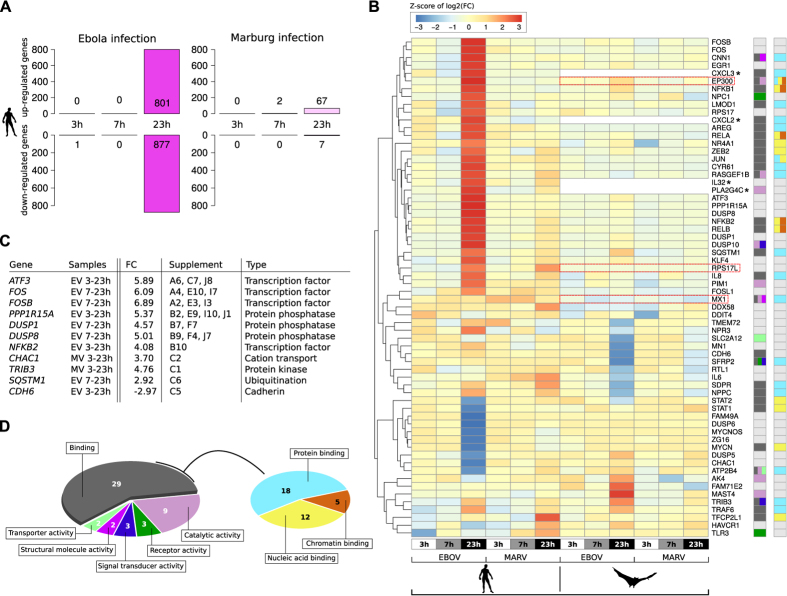
Significantly regulated genes in cells infected with EBOV or MARV. (**A**) Number of strongly regulated human genes after infection with EBOV or MARV. There were only a few genes that were significantly regulated (*padj* <0.1) at 3 and 7 h p.i. in both EBOV- and MARV-infected cells compared with their expression in Mock-treated cells. At 23 h p.i., the number of regulated genes was higher (1,678) in EBOV-infected cells than in cells infected with MARV. Adding these ~1,600 strongly regulated genes to the findings from analyses comparing the different time points or viruses resulted in approximately 2,500 genes being identified as significantly differentially transcribed. (**B**) Heat map of row-scaled log2-fold changes in expression in infected HuH7 and R06E-J-samples against the corresponding Mock samples (e.g., column three shows the fold change between HuH7 Mock-treated cells and HuH7 EBOV-treated cells at 23 h p.i.). The input matrix is scaled within the rows to visualize changes in expression at the gene level. Fold changes are based on unique genome read counts of *H. sapiens* and *R. aegyptiacus*. Genes without a clear homologous sequence in the *R. aegyptiacus* genome or transcriptome assembly are marked with a star. We identified homologous locations (*LOC107508087*, *LOC107515336*, *LOC107498547*) for three genes that were not directly annotated in the *R. aegyptiacus* genome (*EP300*, *RPS17L*, *MX1*). These locations were identified using our *de novo* transcriptome assembly (red boxes). We indicated the molecular function of each gene based on the color scheme presented in (**D**). (**C**) Highly regulated genes in EBOV- and MARV-infected HuH7 and R06E-J cells. FC – log_2_-fold change based on DESeq normalized read counts. See Supplement (Tables S5 and S6) and corresponding entries for detailed information. (**D**) The PANTHER database (v11.0)^96^ was used to assign molecular functions to each of the 64 genes in (**B**) We further subdivided the dominant group of genes that we identified to have a general binding function. During filovirus infections, the most prominent regulatory effects were observed for genes encoding transcription factors, those regulating the *NFκB* and MAPK pathways, their DUSP inhibitors and growth factors (Figure ES2A and Tables S5 and S6, full tables in the electronic supplement). In addition, changes were also observed for genes that regulate protein translation (*RPS17*, *PPP1R15A*), ubiquitination (*TRAF6*, *SQSTM1*), autophagocytosis (*SQSTM1*) and cation transport (*CHAC1*, *ATP2B4*). We also observed the strong up-regulation of genes that are involved in energy transfer (e.g., *RASGEF1B*). Details can be found in Tables S5–S8.

By comparing viral RNA levels in HuH7 and R06E-J cells, we determined that EBOV and MARV replicate more rapidly in HuH7 than in R06E-J cells between 3 and 7 h p.i. RNA synthesis slowed down in both species in the subsequent 16 h (Fig. 3), and transcript levels were nearly identical at 23 h p.i. (Table S2). The EBOV and MARV viral proteins were more abundant in HuH7 than in R06E-J cells at 7 h p.i. and were present at similar levels at 23 h p.i. (Fig. 1C,D) as determined using IFAs. Early increases in the levels of viral RNA and proteins in HuH7 cells may be attributed to a faster rate of early replication of filoviruses in HuH7 cells compared to the rate in R06E-J cells. The differences in replication velocities may contribute to the different susceptibilities of HuH7 cells and R06E-J cells to filovirus infection. However, it does not explain which differences in the cellular regulatory response mechanisms are responsible for the higher RNA synthesis rate in HuH7 cells. We observed few regulatory effects at 3 and 7 h p.i. However, more than 1,670 genes were up- or down-regulated at 23 h p.i. in EBOV-infected HuH7 cells, whereas only 74 genes showed altered expression patterns in MARV-infected samples (Fig. 4A). Chemokine ligands (*CXCL1* and *CXCL5*), transcription factors (e.g., *FOSL1*, *FOS*, *FOSB*, *ATF3*), and genes with diverse functions (e.g., *PPP1R15A*, dual-specificity phosphatases (DUSPs)) were among the 55 genes that were most strongly regulated after filovirus infections in HuH7 cells (log_2_*FC *> 5, Figure ES2A). This finding is supported by previous reports^11^,^14^. Each gene can be viewed along with all relevant details at our webtool: www.rna.uni-jena.de/supplements/filovirus_human_bat/igo.php.

### Differential expression of 2,500 genes

We aimed to answer three questions based on gene transcription levels: (1) What happens during filovirus infections in the host? (2) What are the differences in host-cell responses between EBOV and MARV infections? (3) What are the differences in how HuH7 and R06E-J cells respond after EBOV and MARV infections? Over 2,500 differentially expressed genes were found to be involved in the response to filovirus infections (Tables S4–S10). The most significant differentially expressed genes during the course of both EBOV and MARV infections between HuH7 and R06E-J cells include genes for transcription factors that regulate the expression of kinases and their antagonists and genes involved in ubiquitination processes (Fig. 4B). Although we confirmed the previously reported dysregulation of genes involved in coagulation^11^,^12^,^14^, these were not among the most differentially expressed genes.

With the exception of a few genes, we confirmed previously observed data based on microarray analyses of EBOV-infected HuH7 cells^12^, human macrophages^14^ rhesus monkey cells^13^, and mouse cells^11^.

### Regulation of transcription factors

**Figure 5:**
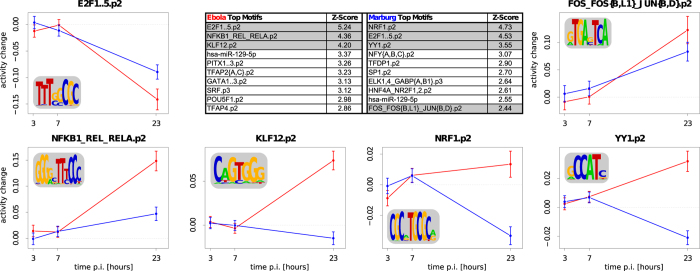
Motif activity response analysis. The table shows the top significant motifs after the infection of HuH7 cells with EBOV (red) or MARV (blue) compared with the response in Mock controls. Regulated motifs are predicted to target (1) the cell cycle (E2F1..5.p2) by down-regulating *CDC6*, *PCNA* and *MCM6*; (2) *NFκB* -signaling (NFKB1_REL_RELA.p2) by targeting CXCL isoforms, *ELF3*, *NFκB* isoforms, *FOSL2* and *JUN*; (3) *EGR1* expression in EBOV-infected cells (KLF12.p2, YY1.p2 and others); or (4) chromatin organization in MARV-infected cells (NRF1.p2, YY1.p2 and others). For selected motifs, the inferred activity changes (points +/−1 SD) after EBOV or MARV infection relative to the corresponding Mock controls are shown for the different time points (3, 7 or 23 h p.i.) adjacent to and below the table. Selected regulatory motifs, the associated genes and their important targets (including their fold change between two time points) can be viewed in Section ES5 and are summarized in File ES5D.

Many transcription factors were found to be among the most differentially expressed genes. Therefore, we assume that they play an important role in the changes observed after filoviral infection. To identify the regulatory factors that are responsible for the observed transcriptional changes after filoviral infection in HuH7 cells, we performed motif activity response analyses with MARA^47^. MARA infers the regulatory impact (also called *activity*) of a regulatory motif from the changes in expression of the predicted downstream genes (targets) of that motif. For a curated collection of ~190 mammalian transcription factor binding motifs and ~90 miRNA seed families, we found 76 and 38 motifs with a change in activity (z-value > 1.5) in response to EBOV and MARV infections, respectively (see electronic supplement, Section ES5). Genes that are regulated by such motifs are involved in *NFκB* signaling or cell-cycle regulation (Fig. 5). Interestingly, we found that the *FOS*/*JUN* -motif has significantly increased activity after both EBOV and MARV infections. This result indicates that genes having *FOS*/*JUN* -motif binding sites in their promoter region are primarily up-regulated (Fig. 5). Consistent with this, transcription factors that are associated with this motif (e.g., *FOSB*) were up-regulated in infected cells (Fig. 4B). The transcription factor *AP1*, a homo or heterodimer of differentially expressed *FOS* and *JUN*, plays important roles in different viral infections^48^,^49^. Other motifs, such as the *KLF12*- and the *NRF1*-associated motifs (Fig. 5), are more specific to EBOV and MARV, respectively, reflecting the differences in the impacts of theses two viruses on the transcriptional landscape of infected cells. In summary, we found various motifs, including the antiviral signaling-associated motif for *NFκB*, to have significant changes in activity (Fig. 5). For each motif, we provide associated regulators and target genes (Section ES5).

### The antiviral mRNA response is mostly unchanged

Unexpectedly, apart from the effect on transcription factors, the majority of genes and pathways that are relevant for viral infections did not demonstrate significant regulatory changes in response to filoviral infection (Section ES6). The majority of genes involved in innate immune responses (from Kuri *et al*.^50^) were either not expressed in the human cell cultures examined here or were only slightly differentially regulated during infection (Table S11). Consistent with this observation, some of these genes (e.g., *IFITM1*/*2*, *OAS1*-*3*) are not included in our bat transcriptome assembly, suggesting they were not transcribed. However, we did observe significantly different RNA levels for several bat homologs of innate immune response genes in filovirus-infected R06E-J cells in comparison with their levels in HuH7 cells. For example, *DDX58* and *ADAR* demonstrated lower, and *NMI* higher, expression levels in EBOV-infected R06E-J cells (Table S11). The differences in the mRNA concentrations of these transcripts in infected HuH7 and R06E-J cells may play a role in the defense mechanisms that are active during filovirus infections. Genes encoding for proteins initiating pathways known to respond to viral infections, such as *DDX58* (Figure ES6.8), *NFκB* (Figures ES6.10 and ES6.17), and MAPK pathways (Figures ES6.2–6.7), were not induced during filovirus infections in our study. Only those genes of the examined pathways that encode proteins that act as key players (see Fig. 4B,C) were up-regulated in EBOV-infected HuH7 cells relative to their expression in R06E-J cells.

The Ebola viral protein VP35 inhibits *DDX58* signaling, which determines the outcome of infection in human cells^51^,^52^,^53^. We inspected the *DDX58* pathway, which induces *ISRE3*, *AP1 (FOS* and *JUN*), *NFκB* and interferon *β* activation in cells^54^,^55^ (Figure ES6.8). Although we observed differences between HuH7 and R06E-J cells in the levels of *DDX58* and *ISYNA1* mRNA, all but one gene (*IKKε*) related to the *DDX58* pathway were expressed, but they were not differentially affected. We noted an up-regulation of the *FOS* (38X) and *JUN* (9X) genes in EBOV-infected HuH7 cells 23 h p.i. (Fig. 4B). We observed no significant change in the genes specific to the *DDX58* pathway at the transcriptomic level. We therefore assume that regulation of the *DDX58* pathway at the transcriptomic level is not a driving factor leading to the frequent fatalities observed in humans, which are not observed in bats, subsequent to filoviral infection.

We investigated the relationship of the *DDX58* protein to other proteins at the mRNA level, such as *TRIM25*, which interacts with the *DDX58* pathway^56^. TRIM-family proteins are induced by interferons and are involved in antiviral cellular responses^57^. We found that several members of the TRIM-family were up-regulated in EBOV-infected cells between 3–7 h p.i. but were down-regulated from 7–23 h p.i. (Table S12). The corresponding bat homologs were either not expressed (e.g., *TRIM71*) or were not significantly differentially expressed between R06E-J and HuH7 cells (e.g., *TRIM25*).

In line with previous reports^58^, our results show that filoviruses neither induce nor block apoptosis (Figure ES6.33), as genes involved in apoptosis were not significantly regulated during EBOV or MARV infections, with the exception of *BBC3*, which was up-regulated 9.6X in EBOV-infected HuH7 cells. However, it is important to note that both cell lines were immortalized, which may explain the minimal effects on genes involved in apoptosis.

### The majority of host genes demonstrate a similar reaction after EBOV and MARV infection

**Figure 6:**
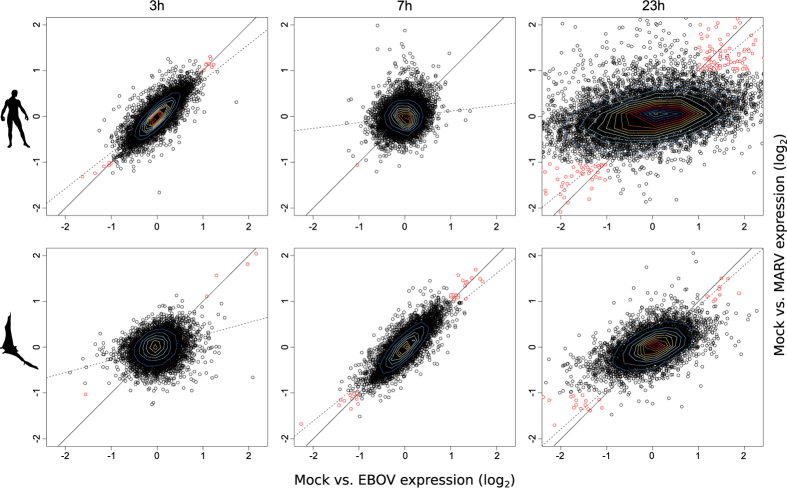
Common gene regulation patterns after filovirus infection. The scatter plots demonstrate the fold changes in expression as determined by DESeq of coding and non-coding RNAs in MARV- and EBOV-infected cells compared with expression in Mock controls 3, 7 and 23 h after EBOV and MARV infections. We observed similar expression patterns in HuH7 cells at 3h p.i. and in the bat cell line 7 h p.i., suggesting that the progress of filovirus infection is slower in R06E-J cells. The scatter plot derived from the differential expression analysis of HuH7 cells at 23 h p.i. shows the large number of differentially expressed genes. A detailed view of the figures (including genes outside of the plotted range of fold changes) can be found in the electronic supplement, Figure ES4B. Genes demonstrating a similar expression after infection with EBOV and MARV and with an *abs* (log_2_ (*FC*)) > 1 are marked in red. Black line: y = x; Dotted line: regression line.

The overall reaction of EBOV- and MARV-infected HuH7 cells at 3 h was similar to the reaction in R06E-J cells at 7 h (Fig. 6). The gene coverage-dependent plot illustrates the greater viral replication velocity in HuH7 cells when compared with the velocity in R06E-J cells. This response may be influenced by viral attachment and entry processes. We investigated the differences and similarities during filovirus infection in HuH7 and R06E-J cells.

#### HuH7

We determined that the majority of the host genes reacted in a similar manner in response to EBOV and MARV infections, which may explain the common symptoms caused by these viruses in humans. *CYR61* was among the most up-regulated genes in HuH7 cells and was usually highly expressed at 3 and 23 h p.i., which correspond to the periods of inflammation and wound repair, respectively^59^ (Figure ES4C). The cytokine genes *IL8* and *IL32* responded in both MARV- and EBOV-infected HuH7 cells, showing a significant up-regulation (Fig. 4B). *IL32* expression can be induced by *IL8* and is involved in the apoptosis of T cells in EBOV-infected patients^60^,^61^. It is also up-regulated in response to influenza A virus infections. The up-regulation of *IL8* results in the activation of pro-inflammatory pathways^62^,^63^. We identified *NRAV* up-regulation 7 h p.i. This long non-coding RNA was recently reported as a key regulator of antiviral innate immunity that acts via the suppression of interferon-stimulated gene transcription^64^. We propose that a cellular component exists, in addition to the filoviral inhibition of the innate immune system by VP24 and VP35^65^,^66^,^67^,^68^,^69^, that results in the same inhibition of innate immunity. At 23 h p.i. we identified several highly up- and down-regulated genes, which we were unable to categorize (see Section ES4). We also determined that *ANXA3* was markedly up-regulated. Annexin A3 is an inhibitor of phospholipase A2 and possesses anti-coagulant properties^70^.

Our data indicate an initial inflammatory response (3 h p.i.)^50^, followed by a repression of antiviral defenses (7 h p.i.) with the majority of up- and down-regulated gene expression occurring at 23 h p.i. in the HuH7 cells (Fig. 6).

#### R06E-J

R06E-J cells responded differently to filovirus infections than HuH7 cells. At 3 h p.i., we identified down-regulations of nuclear receptors involved in cell proliferation and differentiation (e.g., *NR4A3*^71^), and the cell-cycle-regulating ubiquitin ligase *ANAPC10*, which controls the progression through mitosis^72^. *BLCAP*, which controls cell proliferation, apoptosis and the cell cycle^73^, was also down-regulated at 3 h p.i. *HAVCR1* (previously known as *TIM-1*), which was down-regulated at 7 h p.i., is a receptor for many viruses, including filoviruses^74^ and Dengue virus^75^. We observed that various histone genes (e.g., *HIST1H2B6*, *HIST1H1C*) were down-regulated at 23 p.i. This may be an epigenetic signal that could induce cell death. *NPR3* was also down-regulated in R06E-J cells 23 h after filovirus infection. This gene is involved in the regulation of blood volume and pressure, cardiac function and some metabolic and growth processes^76^. Additional information about the significant co-regulation of genes occuring during filovirus infection in HuH7 and R06E-J cells can be found in Section ES4.

These findings may describe the major differences between human and bat cells that occur during filovirus infection.

### Differences in host cell responses after EBOV and MARV infections

**Figure 7:**
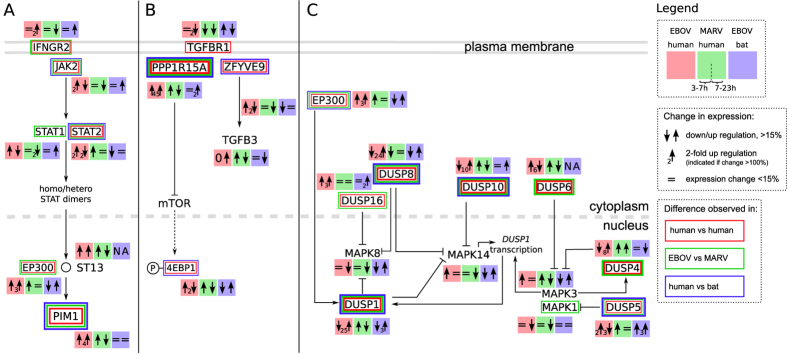
Effects of filovirus infections on JAK/STAT, *PPP1R15A*, and DUSP pathways. **(A) The JAK/STAT pathway.** The JAK/STAT pathway shows a common trend in expression levels: *STAT1*, *STAT2* and *JAK2* were up-regulated (↑) between 3 and 7 h p.i. and then down-regulated (↓) between 7 and 23 h p.i. in EBOV-infected HuH7 cells. The cytokine receptor *IFNGR2* is not regulated between 3 and 7 h (=) and shows a 2X up-regulation between 7 and 23 h (_2_↑) (Figure ES6.22). **(B) The** ***PPP1R15A*** **pathway.** Growth arrest and DNA damage 34 (*GADD34*, officially known as *PPP1R15A*) can be rapidly induced by several types of cellular stress. In R06E-J cells, *PPP1R15A* was slightly up-regulated (2X) due to EBOV infection after 23 h; in HuH7 cells, we observed a strong up-regulation (45X) in EBOV-infected cells and no up-regulation in MARV-infected cells. **(C) The DUSP pathway.** *DUSP1*, *8* and *10* demonstrate the highest specificity for MAPKs (*MAPK14* and *MAPK8*). *DUSP1* is localized in the nucleus, whereas *DUSP8* and *DUSP10* are also available in the cytosol. The nuclear DUSPs are thought to be inducible phosphatases^87^, and the implications of *DUSP*s during viral infections have been demonstrated for *DUSP1*, which is up-regulated during Epstein-Barr virus^97^ and vaccinia virus infections^88^. In response to the vaccinia virus, *DUSP1* is actively involved in antiviral countermeasures of the host cell via the regulation of MAPK phosphorylation. **Legend.** Boxes indicate up/down-regulation from 3 to 7 h and from 7 to 23 h p.i. in EBOV-infected HuH7 cells (red); MARV-infected HuH7 cells (green); and EBOV-infected R06E-J cells (blue). For cases where the expression level changed by more than 15%, an arrow indicates the direction of regulation (↑/↓). When the expression level changed by more than 100% (2-fold change in transcription), the number beside the arrow indicates the fold change. “=” indicates expression changes of <15%. Squares around gene names indicate differential expression within the HuH7 cell line (red), between EBOV- and MARV-infected cells (green) and between HuH7 and R06E-J cells (blue).

Out of the 35 examined pathways (Figures ES6.2–6.33), the JAK/STAT, PPP1R15A and DUSP pathways demonstrated significant differential regulation during infection (Fig. 7). However, these pathways did not demonstrate identical activities in both EBOV and MARV infections, and the responses of these pathways cannot explain the common disease symptoms^9^ induced by both viruses.

#### The JAK/STAT pathway

In EBOV-infected HuH7 cells, all genes coding for members of the JAK/STAT pathway were slightly induced between 3 and 7 h p.i. (Fig. 7A). However, the JAK/STAT system in R06E-J cells demonstrated only a minimal response to EBOV and MARV infections. The EBOV protein VP24 has a negative impact on *STAT1* signaling^77^,^78^,^79^, and *STAT1*/*2* were found to be down-regulated at 23 h compared with their expression at 7 h p.i. in the HuH7 cell line. Downstream genes in this pathway, such as *EP300* and *PIM1*, were highly up-regulated at 23 h at the mRNA level in EBOV-infected HuH7 cells (Fig. 4B). mRNA of the signaling receptor *IFNGR2* was up-regulated, and mRNA of the interacting *JAK2* was down-regulated at 23 h p.i. in EBOV-infected HuH7 cells. This regulation may be attributed to the activation of feedback from PIM1 via CISH to the receptor IFNGR2^80^,^81^ on protein level.

MARV infections trigger a different reaction in HuH7 cells than do EBOV infections: the *PIM1* mRNA and the receptor *IFNGR2* are down-regulated (Fig. 7A). Most strikingly, while we currently do not know whether VP24 also interacts with STAT1 in bats, the complete JAK/STAT pathway is mostly unaffected at the mRNA level during EBOV infections in R06E-J cells (Figure ES6.22).

#### The PPP1R15A pathway

The expression of *PPP1R15A* was markedly up-regulated in EBOV-infected HuH7 cells but only slightly induced in filovirus-infected R06E-J cells (Fig. 4B). We do not currently know how the activity of *PPP1R15A* is linked to EBOV or MARV infections. Figure 7B highlights this extreme differential expression (45X), which suggests that this gene may be a previously unidentified key player in the response of EBOV-infected HuH7 cells.

For MARV-infected HuH7 and R06E-J cells, we did not detect such a huge change in *PPP1R15A* mRNA levels. *PPP1R15A* is involved in the regulation of programmed cell death^82^, and genes involved in apoptosis were not deregulated (Figure ES6.33), which might be because only immortalized cell lines were used in these experiments. Another important activity of *PPP1R15A* is the negative feedback control it exerts on *eIF2α* phosphorylation, which regulates protein translation^83^. *EIF2α* was not significantly differentially expressed in our samples (Figure ES6.12). Protein kinase R, which phosphorylates *eIF2α*, was slightly up- and down-regulated in EBOV- and MARV-infected cells, respectively. It is possible that the previously described inhibition of mTOR by *PPP1R15A*^84^,^85^ is responsible for the fact that the mTOR pathway was not found to be active in the filovirus-infected HuH7 and R06E-J cell lines (Figure ES6.26).

#### The DUSP pathway

The most striking difference between EBOV-infected HuH7 and R06E-J cells was observed among the mRNAs encoding the various DUSPs, which represents a possible key to the contrasting innate immune responses of human and bat cells^86^,^87^ (Fig. 7C). An up-regulated expression of *DUSP1* has been observed for vaccinia virus-infected cells^88^ and EBOV-infected human macrophages^14^. DUSPs are critical regulators of several cellular pathways because they inhibit central immune activator genes such as *MAPK8*, *MAPK14* and *MAPK1*/*3* (also known as *ERK2*/*1*)^87^. While the expression levels of these three immune activator genes did not change significantly p.i., the drastic up-regulation at 23 h p.i. of DUSP genes in HuH7 (up to 25X) but not R06E-J (up to 3X) cells is worth noting.

We hypothesize that the following sequence of events occurs during EBOV infections. Upon EBOV invasion of the host cells, an antiviral response is induced, including the activation of *NFκB* and MAPK. Genes of other innate immune response pathways (e.g., JAK/STAT, *DDX58*) are then suppressed. After EBOV infection, DUSPs are highly up-regulated in HuH7 cells, correlating with the down-regulation of *MAPK8*, *MAPK1*/*3* and *MAPK14* mRNA levels. When translated, these genes are responsible for the innate immune response^89^. *PPP1R15A* plays a central role by binding to the receptor *TGFBR1* and inhibiting additional components of the innate immune system. Compared with HuH7 cells, R06E-J cells demonstrate almost no or only a very slight up-regulation of *PPP1R15A* and DUSPs, very likely leading to a stable antiviral response. Furthermore, the mRNA levels of all JAK/STAT pathway genes in R06E-J cells remain constant during EBOV-infection compared with the levels in HuH7 cells. The viral protein VP24 inhibits *STAT1* activity in humans, blocking signaling into the nucleus. We suggest that a possible feedback loop exists that increases the number of *IFNGR2* receptors in HuH7 cells. The robust expression of interferon-stimulated genes could orchestrate the antiviral response of the infected cell^90^.

### Differences in baseline expression levels between human and bat cell lines

To validate the RNA-Seq-derived read counts and observed differences in the baseline expression levels of certain genes between the HuH7 and R06E-J cell lines, we performed qRT-PCR analyses for the putative EBOV receptors *NPC1*^91^ and *HAVCR1*^92^ and the toll-like receptor *TLR3* on Mock and EBOV samples 3 and 23 h p.i. (Section ES7). We compared the 18S-normalized mRNA levels of these genes (File ES7B) with the RNA-Seq-derived read counts from human and bat cells and found a strong overall correlation. Based on our data, all three genes are expressed in both cell lines. We observed that *NPC1* is clearly more abundant in HuH7 cells than in the *R. aegyptiacus* cell line. *TLR3* is expressed at a greater level in R06E-J cells than in the human cell line, but was also not differentially expressed. These results support our RNA-Seq data. Interestingly, we observed differences in the melting curves for *TLR3* and *HAVCR1* (Figure ES7B), which could be due to differences in the amplified sequences of human and bat RNA as identical degenerate primers were used for the amplification of *TLR3* from HuH7 and R06E-J cells. For *HAVCR1* expression, we observed a slight down-regulation between 3 and 23 h p.i. in the R06E-J cells. However, further studies with different cell lines and primary cells are required.

We observed clear differences in the baseline expression of some genes between the HuH7 and R06E-J cell lines. However, we avoided the complications this issue may cause when comparing homologous human and bat genes by focused on the calculated log_2_-fold changes instead of directly comparing read counts.

### The filovirus infection network

**Figure 8:**
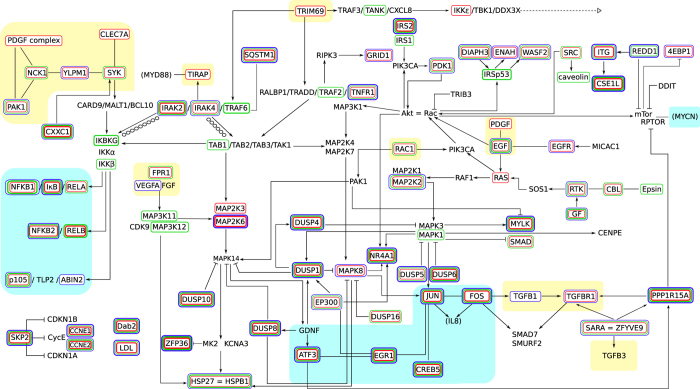
The filovirus infection network. EBOV and MARV keyplayers in HuH7 and R06E-J cells on transcriptional level and their interactions on protein level are displayed. We combined significant differentially expressed genes from known pathways and the literature. Nearly all of the highly deregulated keyplayers of most significant pathways of this study (e.g. MAPK, *NFκB*, JAK/STAT and DUSP) are part of this filovirus infection network. We are not able to connect all genes (e.g. *SKP2* inhibiting *CDKN1B*, *CYCE* and *CDKN1A*). We found seven members of the DUSP pathway (*DUSP1*, *4*, *5*, *6*, *8*, *10* and *16*) being highly deregulated and involved in many interactions, mostly repressing *MAPK8*, *MAPK1*/*MAPK3* and *MAPK14*. We found several transcription factors (e.g. *FOS*, *JUN*, *ATF3*) being up-regulated during EBOV infection on high levels. Cilloniz *et al*. performed global gene expression analysis of spleen samples of mice infected with different mouse-adapted EBOVs^11^. Here, we mainly confirm the results for HuH7 cells. Yellow background—receptors and associated proteins; blue background—nuclear proteins; red boxes—significant differentially expression in HuH7 cells between 3, 7 and 23 h; green boxes—significant differentially expression between EBOV and MARV; blue boxes—significant differentially expression between HuH7 and R06E-J cells. A detailed picture, gene descriptions and gene regulations can be viewed at Figure ES6.1.

In analyzing the protein-level connections among the hundreds of significantly up-regulated genes in relation to the connections at the transcriptional level and in regard to the differences in the analyzed pathways many specific and sometimes suprising relationships between key players were observed. We identified a vast and complex network of interacting host genes that may explain the fatal outcome of the EBOV and MARV infections. We have summarized the genes that are significantly regulated at the mRNA level in the “filovirus infection network” illustrated in Fig. 8. The connections shown are based on known protein/protein interactions from the literature. We summarized the differences between the different time points over the course of infections in HuH7 and R06E-J cells as well as between EBOV and MARV infections. This summary is not complete, because several highly regulated genes, such as *SKP2*, which inhibit *CDKN1B*, *CYCE* and *CDKN1A* were not connected in our filovirus infection network, probably because of missing information in the literature. When investigating other pathways, such as those involving MAPK, *NFκB*, focal adhesion, or *TGFβ*, we observed that the portions of the pathways that were active in the nucleus were differentially regulated between HuH7 and R06E-J cells during filovirus infections.

## Conclusions

The Ebola and Marburg filoviruses cause severe and often fatal infection in humans, whereas bats, shown to be carriers, do not develop disease symptoms after infection. As a first step towards identifying the cellular response that allows bats to survive a filovirus infection, we provide a systematic overview of the genes that are differentially expressed between human and bat cells during EBOV and MARV infections at three time points p.i. Our investigations are based on 18 full transcriptomic datasets.

In addition to the state-of-the-art RNA-Seq data analysis, comprising read counting, normalization and calculations of fold changes, we investigated 1,500 genes (~7% of human genes) in detail, overlapping them with the 2,500 genes potentially affected during filovirus infections. For each gene, we investigated the following aspects: (1) gene synonyms, (2) functional information, (3) different isoforms, (4) characteristics in 5′/3′-UTR, (5) intronic transcripts, (6) single-nucleotide exchanges, (7) ncRNA detection, (8) description of novel genes, (9) genomic context, (10) conservation, (11) expression profile changes, and (12) homologous gene detection in *R. aegyptiacus*. The result of this multidimensional bioinformatics analysis is a comprehensive electronic supplement (www.rna.uni-jena.de/supplements/filovirus_human_bat/igo.php) that provides quick insights into how individual genes of interest are regulated during EBOV and MARV infections via transcriptional changes and the generation of alternatively spliced forms. We were only able to investigate expression patterns in two immortalized cell lines of different tissue origin (humans, liver; bat, embryonic). The data collected and presented here serve as valuable sources of information for generating and testing hypotheses concerning the regulatory circuits that are active in filovirus-infected cells and may accelerate filovirus research.

EBOV and MARV replicate more rapidly in HuH7 cells than in R06E-J cells at the early stages of infection. This behavior is especially distinct for EBOV. This trend was confirmed based on the detection of larger quantities of viral nucleoproteins in HuH7 than in R06E-J cells as assessed via IFA. The slower replication rate of filoviruses in bat cells suggests that they have more time to establish a successful antiviral defense, whereas humans are almost immediately overwhelmed by the virus.

Transcription factors are the most differentially expressed genes in human cells after filovirus infection, and the activity of various transcription factor binding motifs changes after filoviral infection. Some of these changes are common to both filoviruses. However, other changes are specific to EBOV or MARV infections.

We mapped the differential expression data of single genes to known innate immune-response pathways and to those we found significantly enriched. Here, we report pathways that are strongly deregulated between EBOV-infected HuH7 and R06E-J cells. However, expected deregulated pathways, with commonalities in transcriptional responses to EBOV and MARV infections but differences between HuH7 and R06E-J cells, were not observed overall. We condensed the data from this analysis into a single figure representing the filovirus infection network. This network provides valuable insights into previously undescribed interactions and responses to filovirus infections.

Although we examined the expression pattern of three genes (*HAVCR1*, *NPC1*, *TLR3*) by performing qRT-PCR analyses of the HuH7 and R06E-J cell lines, further investigations (e.g., in similar or different cell types, primary cells, or cells of the immune system) are required to improve the compiled data, as are *in vivo* studies on this topic.

We report a systematic and comprehensive computational study that provides a basis for further research into the pathogenesis of filoviruses and contributes significantly to the field of virology in general.

## Additional Information

**How to cite this article**: Hölzer, M. *et al*. Differential transcriptional responses to Ebola and Marburg virus infection in bat and human cells. *Sci. Rep.* **6**, 34589; doi: 10.1038/srep34589 (2016).

## Supplementary Material

Supplementary Information
