# Corrigendum: Differential transcriptional responses to Ebola and Marburg virus infection in bat and human cells

**DOI:** 10.1038/srep39421

**Published:** 2017-01-11

**Authors:** Martin Hölzer, Verena Krähling, Fabian Amman, Emanuel Barth, Stephan H. Bernhart, Victor A. O. Carmelo, Maximilian Collatz, Gero Doose, Florian Eggenhofer, Jan Ewald, Jörg Fallmann, Lasse M. Feldhahn, Markus Fricke, Juliane Gebauer, Andreas J. Gruber, Franziska Hufsky, Henrike Indrischek, Sabina Kanton, Jörg Linde, Nelly Mostajo, Roman Ochsenreiter, Konstantin Riege, Lorena Rivarola-Duarte, Abdullah H. Sahyoun, Sita J. Saunders, Stefan E. Seemann, Andrea Tanzer, Bertram Vogel, Stefanie Wehner, Michael T. Wolfinger, Rolf Backofen, Jan Gorodkin, Ivo Grosse, Ivo Hofacker, Steve Hoffmann, Christoph Kaleta, Peter F. Stadler, Stephan Becker, Manja Marz

Scientific Reports 6: Article number: 3458910.1038/srep34589; published online: 10 07 2016; updated: 01 11 2017

In this Article, Ivo Grosse is incorrectly affiliated to “Department of Soil Ecology, UFZ - Helmholtz Centre for Environmental Research, Theodor-Lieser-Str. 4, 06120, Halle/Saale, Germany”. The correct affiliations for Ivo Grosse are listed below:

Institute of Computer Science, Martin-Luther University Halle-Wittenberg, Von-Seckendorff-Platz 1, 06120, Halle/Saale, Germany.

German Centre for Integrative Biodiversity Research (iDiv) Halle-Jena-Leipzig, Deutscher Platz 5e, 04103, Leipzig, Germany.

