# Supplementary material for: Differential transcriptional responses to Ebola and Marburg virus infection in bat and human cells
Source: Sci Rep. 2016 Oct 7;6:34589. doi: 10.1038/srep34589 (PMC5054393; doi:10.1038/srep34589)
Supplement: Supplementary Information [file srep34589-s1.pdf]

–*Supplementary Data*–

Differential transcriptional responses to Ebola and Marburg virus infection in cells from bats and humans

Martin Hölzer[1,+], Verena Krähling[2,+], Fabian Amman[3], Emanuel Barth[1,27], Stephan H Bernhart[4], Victor AO Carmelo[5,6], Maximilian Collatz[1,2], Gero Doose[4], Florian Eggenhofer[7], Jan Ewald[8], Jörg Fallmann[7], Lasse M Feldhahn[9,10,11], Markus Fricke[1], Juliane Gebauer[8], Andreas J Gruber[12], Franziska Hufsky[1,13], Henrike Indrischek[14], Sabina Kanton[15,16], Jörg Linde[17], Nelly Mostajo B.[1,2], Roman Ochsenreiter[7], Konstantin Riege[1], Lorena Rivarola-Duarte[3,18,19], Abdullah H Sahyoun[1,3,20], Sita J Saunders[21], Stefan E Seemann[5,6], Andrea Tanzer[7], Bertram Vogel[1,2,13], Stefanie Wehner[1,22], Michael T Wolfinger[7,23,24], Rolf Backofen[5,21,25], Jan Gorodkin[5,6], Ivo Grosse[9,10], Ivo Hofacker[5,7], Steve Hoffmann[4], Cristoph Kaleta[8,26], Peter F Stadler[3,5], Stephan Becker[2], and Manja Marz[1,27,*]

[1]RNA Bioinformatics and High Throughput Analysis, Faculty of Mathematics and Computer Science, Friedrich Schiller University Jena, Leutragraben 1, 07743, Jena, Germany
[2]Institute of Virology, Philipps-University Marburg, Hans-Meerwein-Str. 2, 35043 Marburg, Germany
[3]Bioinformatics Group, Department of Computer Science, University of Leipzig, Härtelstraße 16-18, 04107, Leipzig, Germany
[4]Transcriptome Bioinformatics, Junior Research Group, Leipzig Research Center for Civilization Diseases, University of Leipzig, Härtelstraße 16-18, 04107, Leipzig, Germany
[5]Center for Non-coding RNA in Technology and Health, University of Copenhagen, Grønnegårdsvej 3, 1870, Frederiksberg C, Denmark
[6]Department of Veterinary Clinical and Animal Sciences, University of Copenhagen, Grønnegårdsvej 3, 1870, Frederiksberg C, Denmark
[7]Theoretical Biochemistry Group, Institute of Theoretical Chemistry, University of Vienna, Währingerstraße 17, 1090, Vienna, Austria
[8]Research Group Theoretical Systems Biology, Friedrich Schiller University Jena, Leutragraben 1, 07743, Jena, Germany
[9]German Centre for Integrative Biodiversity Research (iDiv) Halle-Jena-Leipzig, Deutscher Platz 5e, 04103, Leipzig, Germany
[10]Institute of Computer Science, Martin-Luther University, Von-Seckendorff-Platz 1, 06120, Halle/Saale, Germany
[11]Department of Soil Ecology, UFZ - Helmholtz Centre for Environmental Research, Theodor-Lieser-Str. 4, 06120, Halle/Saale, Germany
[12]Biozentrum, University of Basel, Klingelbergstraße 50/70, CH-4056, Basel, Switzerland
[13]Chair of Bioinformatics, Faculty of Mathematics and Computer Science, Friedrich Schiller University Jena, Ernst-Abbe-Platz 2, 07743, Jena, Germany
[14]Junior Professorship for Computational EvoDevo, Bioinformatics, Department of Computer Science, University of Leipzig, Härtelstraße 16-18, 04107, Leipzig, Germany
[15]TFome Research Group, Bioinformatics Group, Interdisciplinary Center of Bioinformatics, Department of Computer Science, University of Leipzig, Härtelstraße 16-18, 04107, Leipzig, Germany
[16]Paul-Flechsig-Institute for Brain Research, University of Leipzig, Jahnallee 54, 04109, Leipzig, Germany
[17]Leibniz Institute for Natural Product Research and Infection Biology Hans Knöll Institute (HKI), RG Systems Biology and Bioinformatics, Beutenbergstraße 11a, 07745, Jena, Germany
[18]Max Planck Institute for Mathematics in the Sciences, Leipzig, Germany
[19]Department of Bioanalytical Ecotoxicology, UFZ-Helmholtz Centre for Environmental Research, Leipzig, Germany
[20]Doctoral School of Science and Technology, AZM Center for Biotechnology Research, Lebanese University, Tripoli, Lebanon
[21]Bioinformatics Group, Department of Computer Science, University of Freiburg, Georges-Köhler-Allee 106, 79110, Freiburg, Germany
[22]Institute of Aquaculture, University of Stirling, Stirling FK9 4LA, Scotland, U.K
[23]Center for Integrative Bioinformatics Vienna, Max F. Perutz Laboratories, University of Vienna, Medical University of Vienna, Dr. Bohr-Gasse 9, 1030, Vienna, Austria
[24]Department of Biochemistry and Molecular Cell Biology, Max F. Perutz Laboratories, University of Vienna, Dr. Bohr-Gasse 9, 1030, Vienna, Austria
[25]Centre for Biological Signalling Studies (BIOSS), University of Freiburg, Freiburg, Germany
[26]Research Group Medical Systems Biology, Institute for Experimental Medicine, Christian-Albrechts-University Kiel, Brunswiker Str. 10, 24105, Kiel, Germany
[27]FLI Leibniz Institute for Age Research, Beutenbergstraße 11, 07745 Jena, Germany
[*]Tel: +49 3641 946480; Email: manja@uni-jena.de
[+]these authors contributed equally to this work

Supplementary Table 1: **Read statistics.** Read counts and assembly/mapping statistics for all 18 HiSeq samples and the additional MiSeq library for *R. aegyptiacus*. We mapped all corresponding samples to the *H. sapiens* and *R. aegyptiacus* genome with `TopHat` and `segemehl`. Additionally, we build *de novo* transcriptome assemblies for both species. For *R. aegyptiacus*, a *de novo* transcriptome assembly was computed based on the HiSeq and pooled MiSeq reads. MiSeq data was assembled with `Mira` only. For each assembly tool, the number of contigs (>= 0 *bp*, >= 1000 *bp*) and the N50 value are listed. For `TopHat` and `segemehl`, overall read mapping statistics are provided. A large amount of reads in the EBOV 23 h sample mapped to the EBOV genome. Detailed statistics can be found in the electronic supplement.

Cell line	HuH7 (*Homo sapiens*)									R06E-J (*Rousettus aegyptiacus*)								
Samples	Mock			EBOV			MARV			Mock			EBOV			MARV		
	3 h	7 h	23 h	3 h	7 h	23 h	3 h	7 h	23 h	3 h	7 h	23 h	3 h	7 h	23 h	3 h	7 h	23 h
Read data (million reads)																		
raw	40.5	38.0	39.0	34.4	49.9	53.0	44.2	48.4	36.3	50.4	44.0	48.5	41.5	38.4	39.3	37.5	48.7	45.6
processed	38.4	36.0	36.9	32.8	46.6	50.1	41.8	45.7	34.3	47.8	41.4	45.5	39.4	36.0	37.4	35.5	45.9	43.3
	Mapping on human genome (overall read mapping rate in %)									Mapping on bat genome (overall read mapping rate in %)								
`TopHat`	89.4	90.8	91.3	89.9	88.9	55.7	90.6	89.3	88.9	90.6	90.7	92.2	90.2	91.0	72.4	91.3	91.1	89.1
`segemehl`	95.3	95.6	95.1	95.1	93.1	58.3	95.4	94.5	92.8	97.5	92.0	97.2	97.2	96.7	76.9	97.3	96.9	95.4

Cell line	R06E-J (*Rousettus aegyptiacus*)									
Samples	Mock			EBOV			MARV			pooled
	3 h	7 h	23 h	3 h	7 h	23 h	3 h	7 h	23 h	MiSeq
Read data (million reads)										
raw	50.4	44.0	48.5	41.5	38.4	39.3	37.5	48.7	45.6	38.2
processed	47.8	41.4	45.5	39.4	36.0	37.4	35.5	45.9	43.3	38.0
de novo transcriptome assembly										
	>= 0 *bp*			>= 1000 *bp*			N50			
`Oases`	370 200			180 458			3 875			
`TransABySS`	790 204			169 324			1 788			
`SOAP-Trans`	699 418			147 144			3 261			
`Trinity`	484 826			188 534			5 071			
`Mira`	162 861			21 987			774			
`Combined`	**977 787**			**277 595**			**3 923**			
	Mapping on bat transcriptome (overall read mapping rate in %)									
`TopHat`	94.6	94.7	95.2	94.6	95.0	95.8	95.5	95.2	94.8	–
`segemehl`	98.5	97.0	97.6	98.5	96.6	98.4	98.2	97.6	97.4	–

Supplementary Table 2: **Number of reads mapping to the viral genomes.** For R06E-J samples, we used `Blastn+` to find contigs within the *R. aegyptiacus* transcriptome assembly which represent the full EBOV (contig610) and MARV (contig5818) genome, respectively. Read counts were normalized by library size. Read maximum peaks were calculated for each sample. Interestingly, EBOV seems to replicate much faster in human cells compared to bat cells between 3 and 7 h (15.6X). However, EBOV decreases its transcription speed again in the following 16 h (15.5X) (see Fig. 1B and Fig. 3 in the manuscript). Similarly, MARV replicates faster between 3 and 7 h in human cells (7.6X) than bat cells (4.3X). The RNA profiles mapping to the viral genomes are astonishingly similar, showing no mutations and only a minor fraction of reads mapping to the 5' and 3' UTR of the genome, showing the difference between genomic and transcriptomic level. Read counts are based on unique `TopHat` mappings.

	HuH7 EBOV (KM034562v1)			HuH7 MARV (JN408064)			R06E-J EBOV (contig610)			R06E-J MARV (contig5818)		
	# reads	peak	norm.	# reads	peak	norm.	# reads	peak	norm.	# reads	peak	norm.
Mock 3 h	3 689	182	96.07	134	9	3.49	3 956	124	82.76	158	11	3.31
Mock 7 h	1 897	102	52.69	104	8	2.89	4 722	151	119.85	155	13	3.74
Mock 23 h	3 469	148	94.01	128	6	3.47	4 868	164	106.99	289	10	6.35
EBOV 3 h	28 009	1 653	853.93				39 274	1 156	948.65			
EBOV 7 h	619 370	43 222	13 291.20				162 618	7 260	4 517.17			
EBOV 23 h	10 334 085	429 012	206 269.16				6 853 608	228 449	183 251.55			
MARV 3 h				37 504	1 794	897.22				3 896	126	109.75
MARV 7 h				313 238	13 683	6 854.22				21 654	782	471.76
MARV 23 h				701 757	24 435	20 459.39				848 647	22 119	19 599.24

Supplementary Table 3: **Comparison of genome and *de novo* transcriptome assemblies.** From the genomic sequences of *H. sapiens* and *R. aegyptiacus* we selected different sets of expressed genes using various filter thresholds: 1) we selected transcripts from the genome with at least $N \in \{100, 1000, 5000\}$ unique mapped reads in one sample ($= \exists$) or 2) accumulated all unqiue mapped reads over all samples ($= \sum \forall$). The selected transcript sets were further blasted against the corresponding *de novo* transcriptome assembly of human and bat, respectively. We defined a transcript (derived from the genomic sequence) as true positive and therefore correctly assembled, if we got at least one blast hit with an alignment length > 90%.

	**$\exists$ sample**			**$\sum \forall$ samples**		
**read count**	≥ 100	≥ 1000	≥ 5000	≥ 100	≥ 1000	≥ 5000
H. sapiens	96.54%	97.39%	98.17%	93.0%	97.18%	98.08%
R. aegyptiacus	88.26%	92.8%	94.02%	81.25%	90.20%	92.19%

Supplementary Table 4: **Differential gene expression.** Differential expression levels of `NCBI`-annotated protein-coding genes and ncRNAs, *de novo* `Cufflinks`-predicted genes, and hand-selected genes of interest from the literature. From the 2 364 *de novo* gene loci showing differential expression, 92 % could be mapped back to already annotated genes (hg19 annotations). Thus, we detected 189 (8 %) unannotated gene loci with significant differential expression. DEG – differential expressed genes ((padj) < 0.1); FC – mean fold change of differential expressed genes (top 300 and all) calculated with `DESeq` ($padj < 0.1$); for each gene the maximum fold change obtained over all combinations of time points and infections was used.

	`NCBI`	ncRNAs	*de novo*	genes of interest	Total
HuH7 samples					
# genes	25 051	2 349	18 391	1 508	47 299
# DEG	2 492	20	2 364	167	5 043
FC Top 300	4.74	2.69	4.58	2.53	
standard deviation	± 0.84	± 1.02	± 0.91	± 1.09	
FC total	2.53	2.69	2.35	2.53	
standard deviation	± 1.03	± 1.02	± 1.04	± 1.09	
R06E-J samples					
# genes	11 358	499	10 496	915	23 268
# DEG	641	8	368	58	1 075
FC Top 300	2.28	3.13	5.61	1.64	
standard deviation	± 0.69	1.46	± 2.14	± 0.64	
FC total	1.77	3.13	5.04	1.64	
standard deviation	± 0.68	1.46	± 2.29	± 0.64	
Manually inspected					
	400	117	170	793	1480

Supplementary Table 5: **Top 10 keyplayers of human and bat infection.** Comparison between all conditions and time points within one species. The read_max values are based on multiple mapped reads and candidates listed here are filtered based on a read_max of at least 100 reads in one sample. Fold changes for human samples are based on unique mapped reads. Interestingly, genes coding for histones are up-regulated between 7 h and 23 h in all samples including Mock.

HIST2H4B – in Mock and EBOV highly regulated, probably cell induced, independent from infection; *CENPE* – the other samples are fairly constant at around 500 read_max; superscript sized numbers – among top 10 of following list (sorted by read_max), number of rank; FC – log_2 fold change based on `DESeq` normalized read counts; norm_reads – `DESeq` normalized read counts; change_max – divided read_max values; read_max – maximum number of reads mapping to one nucleotide position of this gene; Mo – Mock; EV – EBOV; MV – MARV; Genes specified by a number refer to the corresponding LOC, for example LOC338651. Further details about differential expression can be obtained from the various tables and pathway figures in the electronic supplement.

	Gene	Samples	FC	norm_reads		change_max	read_max	
EBOV and MARV on human cells (sorted by fold change)								
A1	*PZP*	EV 3 h/23 h	8.38	6.84	2283.13	2.8660	664	1903
A2	*FOSB*[B8]	EV 7 h/23 h	6.89	20.32	2416.03	63.25	4	253
A3	*RPS17*	Mo 3 h/23 h	-6.85	343.00	2.98	-2.0222	1727	854
A4	*FOS*[B3]	EV 7 h/23 h	6.09	36.82	2190.53	63.8333	6	383
A5	*AREG*	EV 7 h/23 h	6.01	1.63	105.27	20.3333	27	549
A6	*ATF3*[B1]	EV 3 h/23 h	5.89	176.81	10457.63	73.7368	19	1401
A7	338651[B4]	EV 3 h/23 h	5.85	10.27	590.85	24.5600	25	614
	read_max	EV 7 h/23 h	5.80	10.57	590.85	34.1111	18	614
A8	*TMEM88*	EV 7 h/23 h	5.74	1.63	86.85	8.6145	83	715
A9	*MYCN*	EV 7 h/23 h	-5.69	3530.65	68.43	-21.2500	340	16
A10	*GZMM*	EV 3 h/23 h	5.67	2.28	104.42	2.28369	141	323
EBOV and MARV on human cells (sorted by read_max)								
B2	*PPP1R15A*	EV 3 h/23 h	5.37	496.95	18529.31	53.1282	39	2072
B5	*EGR1*	EV 3 h/23 h	4.91	136.88	4110.94	31.6154	13	411
B6	*NR4A1*	EV 3 h/23 h	4.44	227.96	4466.49	28.1538	13	366
B7	*DUSP1*	EV 7 h/23 h	4.57	364.87	7569.54	25.3051	59	1493
B9	*DUSP8*	EV 7 h/23 h	5.01	281.28	9032.49	24.0769	26	626
B10	*NFKB2*	EV 3 h/23 h	4.08	443.38	6769.7	23.0000	32	736
EBOV and MARV on bat cells (sorted by fold change)								
C1	*TRIB3*[D2]	MV 3 h-23 h	4.76	8.90	240.54	14.0000	3	42
C2	*CHAC1*[D1]	MV 3 h-23 h	3.70	61.18	796.86	21.9286	14	307
C3	*DDIT4*[D3]	Mo 7 h-23 h	3.66	47.74	609.84	11.4444	9	103
C4	*HIST1H4A*	Mo 7 h-23 h	3.06	40.85	339.59	5.4815	27	148
C5	*CDH6*[D6]	EV 3 h-23 h	-2.97	4279.83	546.64	-6.8868	365	53
C6	*SQSTM1*[D4]	EV 7 h-23 h	2.92	716.19	5435.56	9.4000	80	752
C7	*ATF3*	Mo 3 h-23 h	2.89	28.56	211.33	5.1111	46	9
	read_max	EV 3 h-23 h	2.37	99.81	515.84	5.0800	25	127
C8	*HIST2H4B*[D7]	Mo 7 h-23 h	2.83	49.02	349.36	4.7097	31	146
	read_max	EV 3 h-7 h	2.40	20.14	106.22	6.5556	9	59
C9	*CYP1B1*	MV 3 h-23 h	-2.76	1239.22	182.81	-3.8857	136	35
C10	*DUSP5*[D8]	MV 3 h-23 h	2.74	188.00	1252.58	6.2069	29	180
EBOV and MARV on bat cells (sorted by read_max)								
D5	*PLEKHA4*	EV 7 h-23 h	1.58	76.66	228.41	8.8461	13	115
D9	*MICAL1*	MV 3 h-23 h	1.74	254.74	852.84	5.8824	17	100
D10	*CENPE*	Mo 3 h-23 h	-1.96	30412.24	7837.38	-5.8202	1036	178

Supplementary Table 6: **Top 10 differences between EBOV/Mock, MARV/Mock and EBOV/MARV in human cells.** Genes with highest differential expression between Mock samples and EBOV- and MARV-infected samples, respectively. In addition, genes with the highest differential expression between EBOV- and MARV-infected samples are summarized. By manual inspection, we found *CXCL8*, *AKR1B10* and *AKR1B15* to play rather a minor role, since we determined only low level transcription and mapping artifacts (*AKR1B10* and *AKR1B15* are located next to each other, reads were mapped twice). *AMOTL2* is part of the MAPK signalling pathway (see electronic supplement). LOC100507347 refers to a protein with unknown function (also described as BC078172). Abbreviations as in STab. 5.

	Gene	Samples	FC	norm_reads		change_max	read_max	
Mock vs. EBOV infection (sorted by fold change)								
E1	*ANKRD1*[F1]	23 h	7.41	55.60	9427.27	111.33300	15	1670
E2	*RPS17*	23 h	6.86	2.98	346.09	2.28923	854	1955
E3	*FOSB*	23 h	6.73	22.83	2416.03	63.25	4	253
E4	*PZP*	3 h	-6.45	598.72	6.84	0.71084	472	664
E5	*CXCL8*	23 h	6.40	10.38	754.70	57.0	3	171
E6	*MYCN*	23 h	-6.17	4917.46	68.43	0.04290	373	16
E7	338651[F2]	23 h	6.05	8.94	590.85	55.8182	11	614
E8	*AREG*[F6]	23 h	5.80	1.99	110.54	39.2000	10	392
E9	*PPP1R15A*[F3]	23 h	5.70	414.04	18529.31	51.8000	40	2072
E10	*FOS*	23 h	5.58	52.92	2190.53	25.5333	15	383
Mock vs. EBOV infection (sorted by read_max)								
F4	*DUSP8*	23 h	5.34	222.39	9032.49	48.1538	13	626
F5	*CXCL5*	23 h	5.24	83.40	3158.21	40.6000	10	406
F7	*DUSP1*	23 h	5.34	216.88	7569.54	37.3250	40	1493
F8	*AREG*	23 h	0.00	0.00	105.27	34.3125	16	549
F9	*AMOTL2*	23 h	4.68	914.39	23387.89	33.3171	41	1366
F10	*CREB5*	23 h	4.98	225.37	7117.82	32.2000	10	322
Mock vs. MARV (sorted by fold change)								
G1	*AKR1B10*[H1]	23 h	6.46	34.75	3050.01	65.5714	7	459
G2	*RPS17*	23 h	6.05	2.98	196.81	1.2424	854	106
G3	*AKR1B15*[H4]	23 h	5.77	2.98	163.10	65.5	2	131
G4	*ANXA1*	23 h	5.48	5.96	265.31	0.8041	97	78
G5	*NCF2*	23 h	4.76	17.87	484.96	0.8171	164	134
G6	*CXXC1*[H2]	23 h	4.57	96.30	2282.34	21.0000	19	399
G7	*ANXA3*[H3]	23 h	4.49	63.54	1430.95	19.2222	9	173
G8	100507347[H6]	23 h	4.08	1.99	33.71	8.1500	20	163
G9	*F2RL2*[H7]	23 h	3.86	295.86	4288.50	7.8400	25	196
G10	*CXCL5*[H5]	23 h	3.82	83.40	1179.77	12.2000	10	122
Mock vs. MARV (sorted by read_max)								
H8	*GPX2*	23 h	3.13	124.10	1089.52	6.9118	34	235
H9	*ANKRD1*	23 h	3.79	55.60	770.93	6.3333	15	95
H10	*PTGR1*	23 h	2.66	1551.78	9778.51	4.7797	177	846
EBOV vs. MARV infection (sorted by fold change)								
I1	*PZP*	3 h	7.36	6.84	1124.58	1.4142	664	939
I2	*AKR1B10*[J2]	23 h	7.18	21.05	3050.01	28.6875	16	459
I3	*FOSB*[J4]	23 h	-6.53	2416.03	26.10	-62.25	253	4
I4	*CXXC1*[J5]	23 h	6.12	32.90	2282.34	24.9375	16	399
I5	*AREG*	23 h	-5.60	105.27	2.17	-11.9348	549	46
I6	*GZMM*	23 h	-5.26	82.90	2.17	-2.4030	322	134
I7	*FOS*[J3]	23 h	-5.13	2190.53	67.99	-27.3571	383	14
I8	*GPX2*	23 h	5.11	31.58	1089.52	12.3684	19	235
I9	*F2RL2*	23 h	4.90	143.44	4288.50	12.2500	16	196
I10	*PPP1R15A*	23 h	-4.80	18529.31	719.21	-30.9254	2072	67
EBOV vs. MARV infection (sorted by read_max)								
J1	*PPP1R15A*	23 h	-4.80	18529.31	719.21	-30.9254	2072	67
J6	338651	23 h	-4.28	590.85	30.45	-24.5600	614	25
J7	*DUSP8*	23 h	-4.07	9032.49	537.15	-24.0769	626	26
J8	*ATF3*	23 h	-4.31	10457.63	527.36	-20.9104	1401	67
J9	*ANKRD1*	23 h	-3.61	9427.27	770.93	-17.5789	1670	95
J10	*AREG*	23 h	-3.50	110.54	9.79	-14.5185	392	27

Supplementary Table 7: **Top 15 differences between human and bat cells.** To investigate genes that were differentially expressed between human and *Rousettus aegyptiacus* tissues, we compared *R. aegyptiacus* transcripts with the corresponding human genes. *R. aegyptiacus* transcripts were identified by homology to annotated *Pteropus vampyrus* genes. Most of the top 15 differences between human and bat cells after infection with EBOV and MARV are shut down completely in either human or bat cells. No gene, except *RELN*, is part of STab. 5 or STab. 6, indicating, that these genes are not differentially expressed during infection, but rather point out general differences of the cell lines HuH7 and R06E-J. The genes are associated with calcium regulated pathways (*ATP2B4*), acyl-CoA pathways (*ACADSB*), transcription factors (*HNF4A*), adenylatkinase (*AK4*) possibly for nucleotide synthesis, cell cycle (*CCND2*), keratins for fibrous proteins forming structural framework (*KRT5*, *KRT75*), or are involved in actin pathways (*ACTA2*).
FC – log_2 fold change based on `DESeq` normalized read counts; norm_reads – `DESeq` normalized read counts; EV – EBOV; MV – MARV; Mo – Mock. For the complete table, see the electronic supplement.

Gene	Samples	FC	norm_reads	
			Human cells	Bat cells
Human vs. Bat after EBOV infection				
RELN	23 h	-14.63	32405.90	1.28
ATP2B4	7 h	-13.73	13614.49	0.00
ACADSB	7 h	-13.59	12305.64	0.00
HNF4A	7 h	-13.24	9692.00	0.00
CCND2	23 h	13.18	0.00	9253.02
TRIM71	7 h	-13.07	8592.89	0.00
AK4	7 h	-12.97	8049.84	0.00
ACTA2	7 h	12.95	1.63	12899.06
DAB2	23 h	-12.65	6415.12	0.00
COCH	3 h	-12.54	5956.65	0.00
KRT5	23 h	12.52	0.00	5888.52
KRT75	3 h	12.39	0.00	5369.48
BMP2	23 h	-12.38	5336.06	0.00
SULT1C4	23 h	-12.19	4672.84	0.00
CXCL10	23 h	12.15	0.00	4559.14
Human vs. Bat after MARV infection				
ACTA2	3 h	14.20	0.00	18826.36
ATP2B4	7 h	-13.71	13373.93	0.00
HNF4A	7 h	-13.39	10724.09	0.00
CCND2	23 h	13.38	1.09	11619.63
AK4	23 h	-13.13	8965.17	0.00
RELN	23 h	-13.12	8899.93	0.00
KRT5	23 h	13.12	0.00	8915.03
KRT75	23 h	13.02	1.09	9033.99
TRIM71	7 h	-12.95	7886.39	0.00
ACADSB	23 h	-12.74	5964.10	0.87
MAGED1	3 h	11.86	0.00	3719.89
PTPRZ1	3 h	11.82	0.00	3607.53
COCH	23 h	-10.01	6290.30	6.12
PDPN	7 h	11.02	0.00	2077.09
BMP2	7 h	-11.75	3446.80	0.00

Supplementary Table 8: **Comparison of human and bat cells (EBOV and MARV as replicates) infected with filoviruses (3 h, 23 h).** Although we observed various differences in gene expression profiles between EBOV- and MARV-infected cells, both infections share the same disease symptoms. To find genes that are differentially expressed between human and bat during filovirus infection, we treated EBOV and MARV samples (from the same time point) as replicates for *DESeq* analysis ($padj \leq 0.1$). Genes sorted by the maximum fold change of 3 h and 23 h p.i. More than half of the top 30 genes are related to actin, connecting tissues and cell-cell interaction. Since we observed these massive differences also between human-Mock cells and bat-Mock cells, they might origin from the differences between cell lines HuH7 and R06E-J. To overcome this cell line artifact, we remove differentially expressed Mock samples (between human and bat cells, $padj < 0.1$) and list 30 manually selected genes in STab. 9. Moreover, we used EBOV and MARV samples at same time points as replicates to analyze the impact of filovirus infection compared to Mock in the human cell line (STab. 10).
FC – log_2 fold change based on `DESeq` normalized read counts; norm_reads – `DESeq` normalized read counts; read_max maximum number of reads mapping to one nucleotide position of this gene; EV – EBOV; MV – MARV. For the complete table, see the electronic supplement.
Genes related to actin, connecting tissues and cell-cell interaction are marked.

			norm_reads		read_max				
			human	bat	human		bat		
Gene	Sample	FC_{max}	EV+MV	EV+MV	EV	MV	EV	MV	Function
COL5A1	23 h	16.39	0.42	36585.11	0	2	904	1288	connective tissues
ATP1A3	23 h	16.25	0.48	37903.82	1	0	2524	2370	cation Na^+/K^+ transport
ACTA1	23 h	15.82	1.45	84020.59	2	0	15143	18700	actin, alpha skeletal muscle
COL6A3	23 h	15.26	0.42	16721.46	1	1	307	369	connective tissues
EEF1A2	3 h	15.15	6.1	221259.05	2	3	31523	30185	Elongation factor 1-alpha 2
CCND2	23 h	14.96	0.42	13558.56	0	1	1790	2633	cell cyclus
MYO10	3 h	14.45	0.34	7571.73	24	37	285	178	actin-based, filopodia
RELN	23 h	-14.19	15418.01	0.82	502	234	1	0	cell-cell interaction
ACADL	3 h	13.91	0.34	5231.1	0	1	567	492	Acyl CoA
PTK7	23 h	13.7	1.33	17812.49	1	1	694	930	tyrosin protein kinase
COL4A2	3 h	13.7	0.34	4529.52	0	1	156	108	connective tissues
GPM6A	23 h	13.4	0.42	4589.59	1	2	877	942	membrane glycoprotein
KRT75	23 h	13.39	0.91	9771.54	12	9	904	1621	extracellular matrix
MAP3K13	23 h	-13.32	11719.28	1.15	2743	2328	1	2	serine/threonine kinase
ACTA2	3 h	13.22	2.64	25179.38	10	12	2882	3400	actin, alpha smooth muscle
RASA3	3 h	13.22	0.44	4196.36	2	0	303	202	GTPase activating
ACTG2	23 h	13.13	1.27	11391.44	0	2	1112	1901	actin, cytoskeleton
KIT	23 h	13.12	0.48	4306.14	1	0	214	221	cytokin receptor
PXDN	23 h	13.11	0.97	8596.75	5	2	320	625	peroxidasin homolog
ADAM12	3 h	13.11	0.88	7802.54	3	7	395	396	cell-cell ineraction
SPG20	23 h	13.06	0.42	3621.9	1	1	230	265	microtubulin, GTP
CACNA2D1	3 h	13.0	0.44	3615.05	9	6	191	184	Ca^{2+} channel complex
LOXL1	3 h	12.9	0.88	6733.85	2	0	529	464	connective tissues
HTR1D	3 h	12.89	0.44	3351.02	1	0	322	496	serotonin rexeptor
PTPN13	3 h	12.88	2.0	15025.71	10	12	418	312	cytoskeleton, GTPase
SLC26A5	3 h	-12.77	4089.95	0.59	1085	1480	1	1	prestin, motor protein
IQGAP2	3 h	-12.72	7910.54	1.17	440	500	2	1	*Ras*-GTPase
COL1A1	3 h	12.72	14.31	96443.9	4	4	3277	1784	connective tissues
TMEM47	3 h	12.72	0.34	2285.25	0	1	377	529	transmembrane protein
KCNA4	3 h	12.69	1.02	6731.18	1	2	333	251	hexokinase

Supplementary Table 9: **Comparison of human and bat samples (EBOV and MARV as replicates) with filovirus infected samples (3 h, 23 h).** To find genes that are differentially expressed between human and bat during filovirus infection, we treated EBOV and MARV samples (from the same time point) as replicates for *DESeq* analysis ($padj \leq 0.1$). We reduced the influence of the different cell types by removing all genes from the initial list (STab. 8) which were also detected as significantly differentially expressed between Mock samples ($\text{Mock}_{3h,7h,23h}$ used as replicates for human and bat samples, respectively). Examples in this list are manually selected from both lists. Genes sorted by the maximum fold change of 3 h and 23 h p.i..
Rk – Rank/position in the corresponding sample list. Abbreviations as in STab. 8. For the complete table, see the electronic supplement.

				norm_reads		read_max				
				human	bat	human		bat		
Gene	Sample	Rk	FC_{max}	EV+MV	EV+MV	EV	MV	EV	MV	Function
ALPK3	23 h	1	-5.94	3121.47	50.99	177	58	11	5	kinase, adenovirus related
ARHGAP20	23 h	2	5.82	0.85	48.12	16	12	15	8	GTPase activated protein
SCN4A	23 h	3	5.17	1.7	61.25	2	2	10	14	sodium channel
TCTEX1D4	3 h	1	4.94	0.44	13.51	10	2	4	5	connecting phosphatase
OSGIN1	3 h	2	-4.47	513.5	23.1	58	176	12	44	oxidative stress, inhibits growth
SLC12A3	23 h	4	4.3	14.62	287.7	71	145	20	40	sodium chlorid carrier
SLC16A11	23 h	5	4.29	1.39	27.19	3	2	6	6	carrier monocarboxylate
CCDC78	23 h	6	-4.23	30.8	1.65	6	3	1	2	unknown function
IGSF6	23 h	7	-4.22	30.75	1.65	5	5	3	2	immunoglobulin, inflammatory
UNC13A	23 h	8	4.17	0.97	17.41	7	9	11	8	vesicle, exocytose
NEIL1	23 h	9	-3.9	42.93	2.87	6	5	5	4	endonuclease, modulated by virus
METRN	3 h	4	-3.85	31.13	2.16	11	11	4	14	cell differentiation
ELN	23 h	10	3.79	0.97	13.37	3	2	4	6	elastin, cell-cell
SLC40A1	23 h	11	-3.78	6409.48	465.85	755	1770	59	82	carrier, iron
C11orf52	23 h	12	-3.73	22.87	1.72	10	10	1	3	together with HSP transcribed
SLC10A1	23 h	13	-3.73	26.07	1.97	5	3	5	4	carrier, NA^{2+}, entry point HBV/HDV
IGSF6	3 h	5	-3.72	19.03	1.44	5	5	3	2	immunoglobulin
MAP6	23 h	15	3.56	0.97	11.48	13	3	6	6	microtubule associated protein
TMEM27	23 h	16	-3.55	23.1	1.97	29	14	4	3	transmembrane
TMOD4	23 h	17	3.53	10.37	119.62	4	6	17	29	tropomodulin, related muscle actin
GRIN2D	23 h	19	-3.25	63.38	6.66	10	7	5	5	glutamate receptor
CLEC4A	23 h	20	-3.24	24.01	2.54	6	9	7	4	cell-cell, immune system
UBC	23 h	24	-3.14	31161.4	3539.27	12040	4245	774	554	ubiquitin
CEP72	23 h	25	-3.11	1003.72	116.45	115	52	21	23	microtubuli, centromer
MAST4	23 h	26	3.11	602.04	5186.53	31	44	168	84	microtubuli
ELF3	23 h	27	-3.1	1012.45	118.16	218	66	19	30	TF, effector of ERBB2 pathway
GLDN	23 h	28	-3.06	28.62	3.44	7	7	21	5	Ranvier nodes along muelinated axons
TRAF4	3 h	15	-2.32	1743.78	348.73	731	201	97	136	activation of $NF\kappa B$ + MAPKs
PLIN2	23 h	54	-2.24	4646.53	983.85	1199	473	152	209	lipid storage
TRIB1	3 h	17	-2.18	909.52	201.26	296	147	118	70	Ser/Thr protein kinase

Supplementary Table 10: **Comparison of filovirus infection to Mock samples (EBOV and MARV as replicates).** Comparison of filovirus (EBOV and MARV treated as replicates) infected samples at 23 h p.i. against Mock samples (3 h, 7 h and 23 h treated as replicates) of human cell samples ($padj < 0.1$), to find genes differentially expressed in both filovirus-infected cells compared to Mock. Genes sorted by the maximum fold change and filtered manually for interesting hits. Abbreviations as in STab. 9. For the complete table, see the electronic supplement.

			norm_reads		read_max			
Gene	Rank	FC_{max}	$MO_{3,7,23}$	$EV_{23}+MV_{23}$	MO_{read_max}	EV_{read_max}	MV_{read_max}	Function
SBK3	1	4.68	2.02	51.73	2	11	6	kinase
SULT1E1	2	-4.56	25.56	1.09	6	7	4	sulfotransferase
PLAU	4	-3.91	175.46	11.7	33	29	23	urokinase, degra. of ex. matrix
FMNL1	8	3.67	35.67	455.05	5	47	25	cytokinese
ANXA3	20	2.79	226.65	1562.27	66	296	173	cell growth
MYCNOS	21	-2.74	296.44	44.27	73	57	75	viral related oncogene
MYCN	31	-2.44	3825.08	702.93	373	340	322	transcription factor
CYP1A1	32	-2.44	2652.15	489.17	236	395	562	cytochrome p450, electron
GDF15	43	2.24	404.62	1915.81	84	431	332	cell growth, inflammation
PEG10	53	-2.09	148275.9	34921.14	6192	7865	6568	retrotransposon-derived protein
SKP2	66	-1.92	14370.05	3798.2	1621	1233	1536	s-phase kinase-associated

Supplementary Table 11: **Expression of genes involved in IFN-induction and -signaling.** The IFN signaling pathway and the induced antiviral effector proteins are important antiviral defence mechanisms[1]. We checked the expression of genes involved in IFN signal transduction, immune/antiviral response and ISGylation for differential expression during EBOV and MARV infection. We found many genes to be not expressed (*IFIH1*, *IRF7*, *GBP1*, *IFI16*, *IFI27*, *IFI35*, *IFI44*, *IFI44L*, *IFIITM1*, *IFITM2*, *OAS1*, *OAS2*, *OAS3*, *OASL*, *TRIM21* and *HERC6*). However, several genes were up-regulated between 3 h and 7 h p.i. and down-regulated between 7 h and 23 h p.i. (*STAT-1*, *STAT-2*, *ADAR*, *IFIT1*, *IFIT5*, *MX1*, *MX2*, *TRIM22*, *TRIM25*, *UBE2L6* and *USP18*). Listed genes were selected according to Weber *et al.*[1]. First characters refer to the expression between 3 h and 7 h p.i., second characters to the expression between 7 h and 23 h p.i. Numbers correspond to the read maximum of the sample. ↑ – up-regulated; ↓ – down-regulated; = – equal expression; 0 – no expression. Numbers preceding arrows indicate up-/down-regulation for more than 200 % (2 – 200 %, 3 – 300 % and so on).

Category/gene	Human						Bat					
	MOCK		EBOV		MARV		MOCK		EBOV		MARV	
**IFN signal transduction**												
DDX58 (RIG-I)	=↑	112	↑↑	156	=↑	133	00	4	00	8	${}_{4}\uparrow\downarrow$	11
IFIH1 (MDA5)	00	–	00	–	00	–	NA	–	NA	–	NA	–
IRF7	00	–	00	–	00	–	NA	–	NA	–	NA	–
IRF9	↑=	53	↑↑	123	↑↓	74	NA	–	NA	–	NA	–
NMI	$\uparrow_{2}\downarrow$	24	==	18	=↓	21	↓↓	244	↓=	179	==	184
STAT-1	=↓	343	↑↓	389	$=_{2}\downarrow$	433	==	200	=↑	231	↑=	250
STAT-2	↑=	76	${}_{2}\uparrow_{2}\downarrow$	88	↑=	79	==	74	↓=	50	↑=	84
STAT-3	↑↓	166	↑=	203	↑↓	183	==	242	=↑	318	↑=	256
**Immune/antiviral response**												
ADAR	==	1041	$\uparrow_{2}\downarrow$	1320	=↓	1329	↓=	164	↓↑	118	↑↑	150
EIF2AK2 (PKR)	↓↓	122	=↑	107	=↓	111	NA	–	NA	–	NA	–
GBP1	00	–	00	–	00	–	NA	–	NA	–	NA	–
IFI16	00	–	00	–	00	–	NA	–	NA	–	NA	–
IFI27	00	–	00	–	00	–	NA	–	NA	–	NA	–
IFI35	↓=	199	=↑	275	↑=	325	↑0	16	↑↓	11	↑↑	15
IFI44	00	–	00	–	00	–	NA	–	NA	–	NA	–
IFI44L	00	–	00	–	00	–	NA	–	NA	–	NA	–
IFIT1	↑↑	260	$\uparrow_{4}\downarrow$	245	↑↓	273	NA	–	NA	–	NA	–
IFIT5	↓↓	59	$=_{3}\downarrow$	41	=↓	55	NA	–	NA	–	NA	–
IFITM1	00	–	00	–	00	–	NA	–	NA	–	NA	–
IFITM2	00	–	00	–	00	–	NA	–	NA	–	NA	–
IFITM3	=↓	15	$0_{3}\uparrow$	24	=↓	14	NA	–	NA	–	NA	–
MX1	00	–	↑↓	22	$\uparrow_{2}\downarrow$	22	=↑	150	↓↑	159	${}_{2}\uparrow\uparrow$	268
MX2	↑=	241	↑↓	271	↑↓	343	↑=	134	↓↑	156	${}_{2}\uparrow\uparrow$	256
OAS1	00	–	00	–	00	–	NA	–	NA	–	NA	–
OAS2	00	–	00	–	00	–	00	–	00	–	00	–
OAS3	00	–	00	–	00	–	NA	–	NA	–	NA	–
OASL	00	–	00	–	00	–	↓2=	15	00	9	↑↑	19
PLSCR1	↓↑	51	↑↓	47	↑=	48	NA	–	NA	–	NA	–
RSAD2 (Cig5)	00	–	00	–	00	–	00	–	00	–	00	–
SP100	${}_{2}\uparrow=$	26	↑↑	44	==	34	↓↑	84	=↑	93	↑↑	115
PMP22	↑↓	15	↑↑	16	=↓	17	NA	–	NA	–	NA	–
**Ubiquitylation and ISGylation**												
HERC5	↓=	14	↑=	16	==	11	↓↑	24	↓↑	23	↑=	23
HERC6	00	–	00	–	00	–	↓↓	45	↓=	46	==	40
ISG15	00	–	$=_{4}\uparrow$	36	0=	11	NA	–	NA	–	NA	–
UBE2L6	↑=	89	↑↓	76	$\uparrow_{3}\downarrow$	117	↓↑	20	↑↓	22	${}_{3}\downarrow_{3}\uparrow$	24
USP18	↑=	70	$\uparrow_{2}\downarrow$	106	↑↓	77	NA	–	NA	–	NA	–

Supplementary Table 12: **The most regulated TRIM genes.** TRIM proteins were recently reviewed by Ozato *et al.*[2]. They represent a superfamily of tripartite motif-containing proteins with more than 60 members from which several are known to be required for the restriction of lentivirus infections. Based on their emerging role in innate immunity, we investigated their features. We identified at least 11 TRIM genes (*TRIM2*, *6*, *8*, *15*, *16L*, *25*, *32*, *34*, *38*, *45*, *47*, *54*, *67*, *71*) to be differentially regulated. *TRIM14*, *21* and *22* were not reported to be differentially expressed, but show interesting features in a small level of transcripts (see electronic supplement). Classical fold change values are reported in the electronic supplement. EV – EBOV; hum – human; read_max – maximum number of reads mapping to one nucleotide position of this gene.

		read_max			
TRIM	Sample	3 h	7 h	23 h	Remarks
TRIM2	hum-EV	143	184	164	*TRIM2* localizes to cytoplasmic filaments
	bat-EV	106	99	110	
TRIM6	hum-EV	85	104	57	Down-regulation for EBOV 23 h, a read-through transcript from this gene into the downstream *TRIM34* gene has been observed, which is here not the case
	bat-EV	NA	NA	NA	
TRIM8	hum-EV	120	116	383	*TRIM8* localizes to nuclear bodies; strong up-regulation for EBOV 23 h
	bat-EV	437	648	523	
TRIM14	hum-EV	107	160	223	
	bat-EV	15	23	24	
TRIM15	hum-EV	10	12	33	*TRIM15* localizes to the cytoplasm
	bat-EV	NA	NA	NA	
TRIM16L	hum-EV	32	26	15	
	bat-EV	109	119	200	putative homolog
TRIM21	hum-EV	<10	<10	<10	
	bat-EV	45	48	62	
TRIM22	hum-EV	84	160	83	
	bat-EV	60	68	73	
TRIM25	hum-EV	80	103	26	*TRIM25* localizes to the cytoplasm; interacts with *DDX58*; similar pattern after MARV infection, containing mir-3614 in 3'UTR
	bat-EV	319	255	299	a much higher and constant level of transcription than human cells
TRIM32	hum-EV	65	63	34	*TRIM32* localizes to cytoplasmic bodies; Mock 23 h & EBOV 23 h down-regulated, MARV 23 h up-regulated (read_max:142)
	bat-EV	128	111	120	
TRIM34	hum-EV	9	13	11	here no read-through transcript from the upstream *TRIM6* gene
	bat-EV	NA	NA	NA	
TRIM38	hum-EV	14	15	14	almost no expression
	bat-EV	NA	NA	NA	
TRIM45	hum-EV	11	19	15	*TRIM45* may function as a transcriptional repressor of the mitogen-activated protein kinase pathway almost no expression
	bat-EV	46	30	54	putative homolog
TRIM47	hum-EV	12	18	17	almost no expression
	bat-EV	26	23	25	putative homolog
TRIM54	hum-EV	0	0	0	may be important for the regulation of titin kinase and microtubule-dependent signal pathways in striatedmuscles; no expression
	bat-EV	NA	NA	NA	
TRIM67	hum-EV	17	39	41	up-regulated in EBOV 7 h
	bat-EV	NA	NA	NA	
TRIM69	hum-EV	10	14	18	Only the first two exons are transcribed, possibly a splice variant
	bat-EV	NA	NA	NA	No homolog in Pva and Rae
TRIM71	hum-EV	506	860	283	E3 ubiquitin protein ligase; MARV-infected cells stay at about read_max=750
	bat-EV	0	0	0	

Supplementary Table 13: **Novel genes related to filovirus infection.** During our analysis we detected several hundreds of novel genomic positions in the human genome being highly differentially expressed during infection with EBOV. Here we list some novel detected genomic locations, highly differentially expressed in human cells. For a detailed view of novel genomic locations detected by `Cufflinks`, see the electronic supplement.

Chromosome	Start	End	ID
chr8	81,451,919	81,455,328	XLOC_016935
chr19	45,972,675	45,973,389	XLOC_009107
chr6	155,282,269	155,284,746	XLOC_15377
chr19	564,029	565,599	XLOC_008950
chr18	56,113,047	56,118,281	XLOC_007908
chr15	23,265,433	23,267,219	XLOC_005909
chr9	68,429,835	68,430,526	XLOC_017600
chr11	46,450,144	46,450,791	AMBRA1 intronic transcript

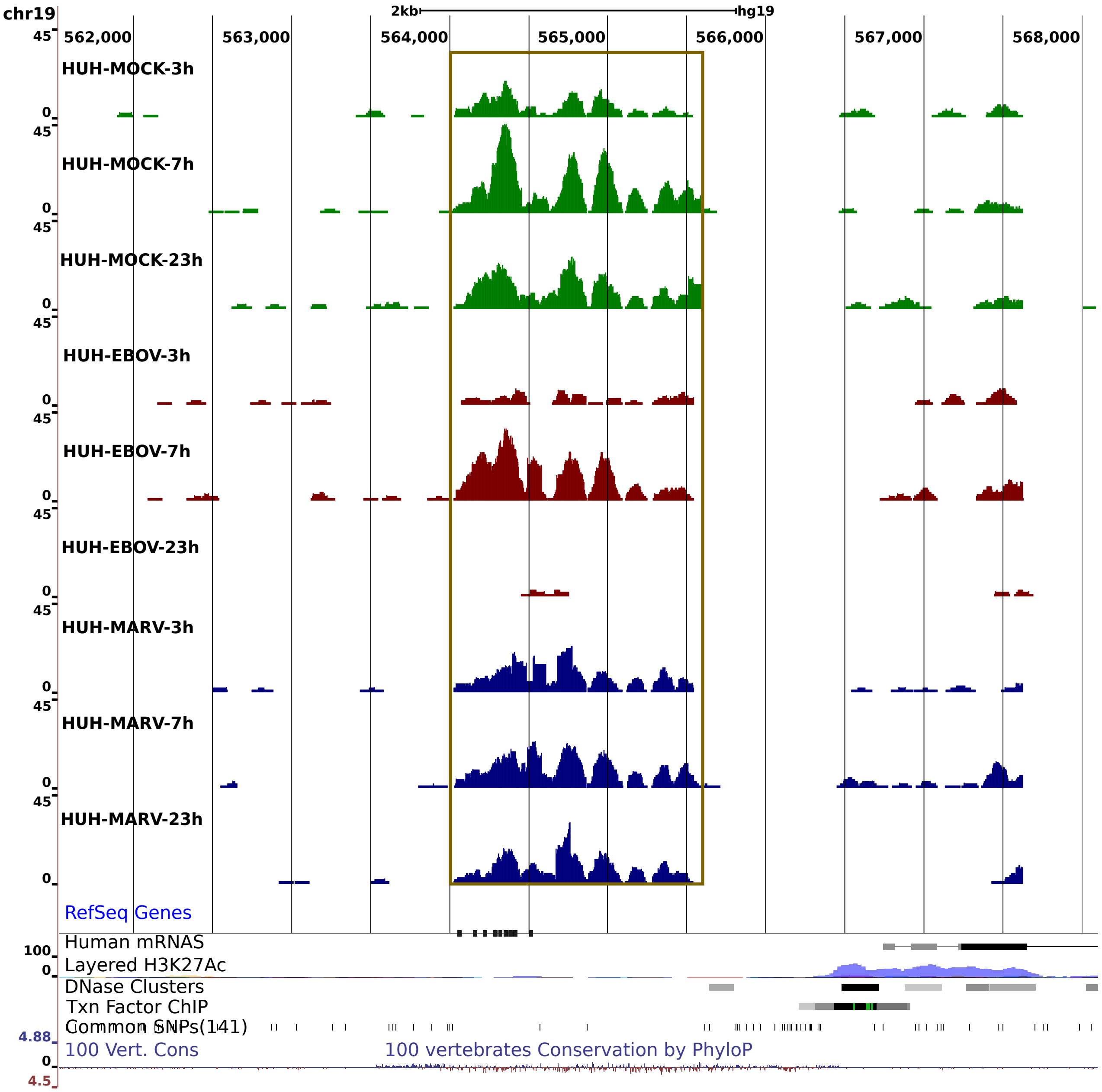

Supplementary Figure 1: **A novel gene.** UCSC screen of XLOC_008950 which encodes a putative novel ncRNA (framed locus). The transcription tracks show moderate expression of this locus in the human Mock and MARV-infected cells (green and blue, respectively). For EBOV-infected cells (red), expression was not detectable at 23 h p.i..

Non-coding RNAs

We investigated 103 human annotated ncRNAs manually. `DESeq` identified 20 of them, to be differentially expressed. From these 20 ncRNAs, more than three quarters are miRNAs (11) and snoRNAs (5) followed by three antisense RNAs of UCKL1, CDKN2B, AFAP1. A manual inspection refused the antisense RNAs to play a major role in EBOV and MARV infection.

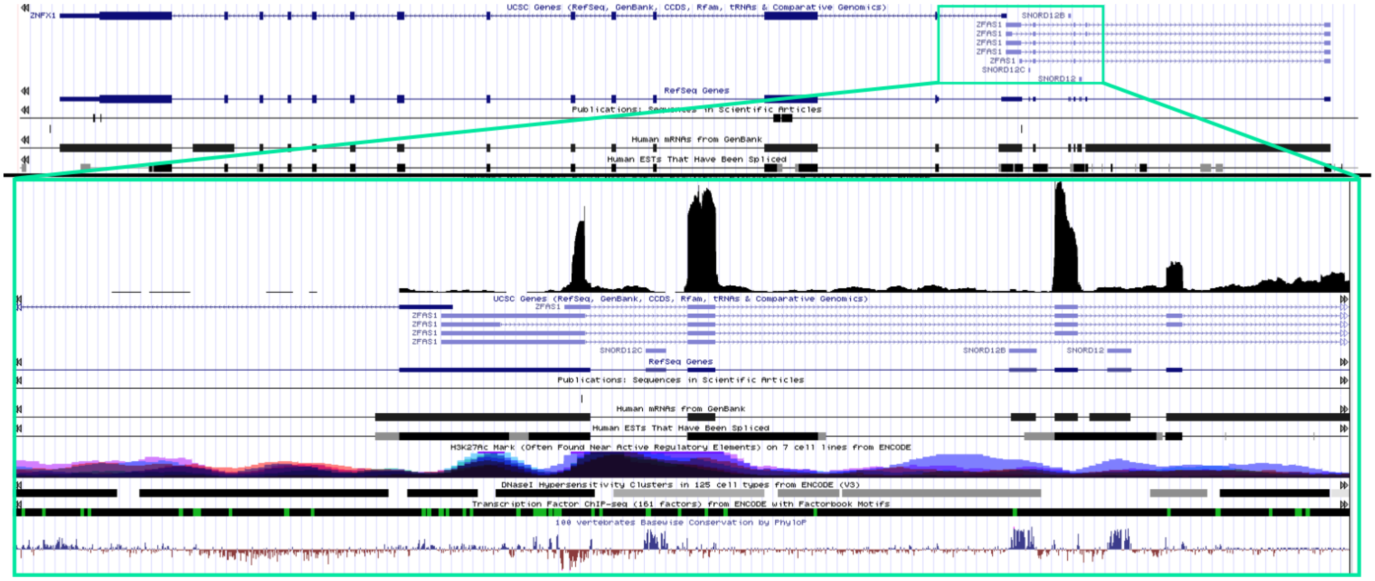

Supplementary Figure 2: **The *ZNFX1* and *ZFAS* genes.** UCSC screenshot of *ZNFX1* and *ZFAS* regulated by a bidirectional promoter. The *ZNFX1* antisense RNA 1 (*ZFAS1*) is expressed and possibly a regulator of *ZNFX1* [3]. For MARV infection, a lower expression level in the 23 h sample could be observed. Remarkable is the bidirectional promoter for the *ZNFX1* gene and the antisense RNA (asRNA) itself.

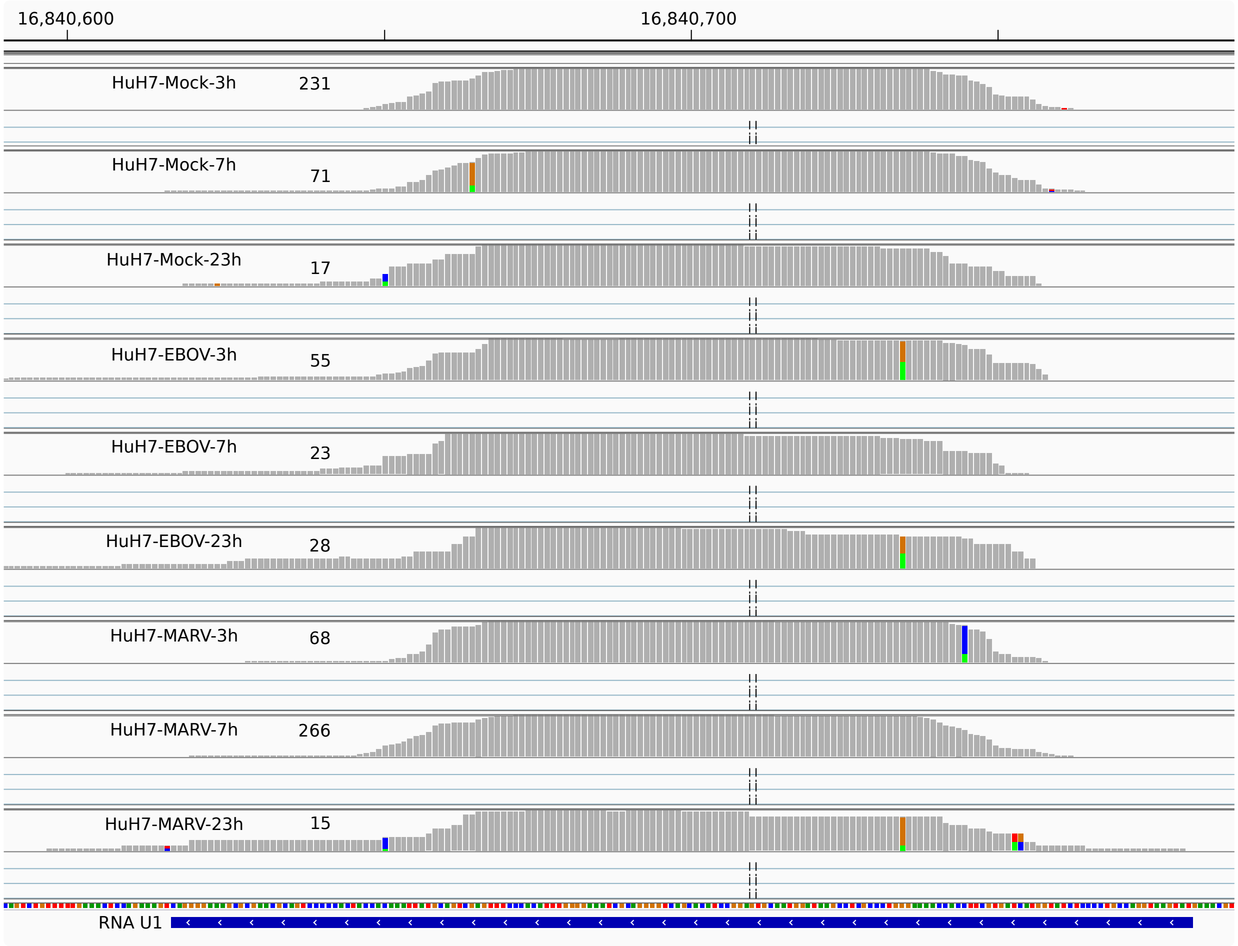

Supplementary Figure 3: **The U1 snRNA gene.** IGV screenshot of RNA U1 small nuclear 1, which is an essential component of the spliceosome as it is responsible for pre-mRNA splicing. The 5' end of this snRNA base pairs with the 5' splice site . Further snRNAs (U2, U4, U5 and U6) are needed the remove the intron and ligate the exons [4]. We observed differential expression for the gene encoding the U1 snRNA. Most transcripts (266 reads) were detected in the 7 h sample of cells infected with MARV, whereas for the 23 h sample only 15 reads were mapped. High expression was also found for the Mock-3 h cells (231 reads). For the other time points of the wildtype less transcripts were observed (71 & 17 reads). Furthermore there were some single nucleotide mutations. For the 3 h EBOV, 23 h EBOV and 23 h MARV sample the Guanine at 16,840,734 was replaced by Adenine (G-to-A:46%,46%,17%). For the 7 h Mock in 22% of the 55 reads the Guanine was replaced by an Adenine at 16,840,734, in the 3 h MARV sample at position 16,840,744 and 25% of the 60 mapped transcripts had an Adenine instead of Cytosine. In the electronic supplement, we report differences between samples even on nucleotide level.

Supplementary Table 14: **Overview of the significantly enriched pathways during EBOV and MARV infection.** Down-regulated (left) and up-regulated (right) pathways for the conditions Mock versus EBOV and Mock versus MARV for the time points 3 h, 7 h and 23 h. All pathways are enriched in at least three samples. For each pathway only the time points with significant changes together with their corresponding adjusted p-values are listed. The different gray scales represent the significance level of the p-values, from 0.1 (light), 0.05 (middle) and 0.001 (dark).

Pathway	Enriched samples (×0.01)											
	down-regulated						up-regulated					
	EBOV			MARV			EBOV			MARV		
	3h	7h	23h	3h	7h	23h	3h	7h	23h	3h	7h	23h
MAPK signaling pathway				0.0308					0.0002		0.0108	0.0357
Focal adhesion				0.0495					0.0078		0.0309	0.0357
Complement and coagulation cascades			0.0043	0.0308	0.0131	0.0015	0.0207					
Cell cycle		0.0001	0.0383			0.0029						
Peroxisome			0.0108		0.0049	0.0115						
Steroid hormone biosynthsesis							0.0059				0.0433	0.0213

References

[1] Kuri, T. *et al.* Interferon priming enables cells to partially overturn the SARS coronavirus-induced block in innate immune activation. *J. Gen. Virol.* **90**, 2686–2694 (2009).

[2] Ozato, K., Shin, D. M., Chang, T. H. & Morse, H. C. TRIM family proteins and their emerging roles in innate immunity. *Nat. Rev. Immunol.* **8**, 849–860 (2008).

[3] Askarian-Amiri, M. E. *et al.* SNORD-host RNA Zfas1 is a regulator of mammary development and a potential marker for breast cancer. *RNA* **17**, 878–891 (2011).

[4] O'Reilly, D. *et al.* Differentially expressed, variant U1 snRNAs regulate gene expression in human cells. *Genome Res* **23**, 281–291 (2013). URL `http://www.hubmed.org/display.cgi?uids=23070852`.
